# Correlation of Influenza Virus Excess Mortality with Antigenic Variation: Application to Rapid Estimation of Influenza Mortality Burden

**DOI:** 10.1371/journal.pcbi.1000882

**Published:** 2010-08-12

**Authors:** Aiping Wu, Yousong Peng, Xiangjun Du, Yuelong Shu, Taijiao Jiang

**Affiliations:** 1 National Laboratory of Biomacromolecules, Institute of Biophysics, Chinese Academy of Sciences, Beijing, China; 2 Graduate School of the Chinese Academy of Sciences, Beijing, China; 3 State Key Laboratory for Molecular Virology and Genetic Engineering, National Institute for Viral Infectious Disease Control and Prevention, Chinese Center for Disease Control and Prevention, Beijing, China; Imperial College London, United Kingdom

## Abstract

The variants of human influenza virus have caused, and continue to cause, substantial morbidity and mortality. Timely and accurate assessment of their impact on human death is invaluable for influenza planning but presents a substantial challenge, as current approaches rely mostly on intensive and unbiased influenza surveillance. In this study, by proposing a novel host-virus interaction model, we have established a positive correlation between the excess mortalities caused by viral strains of distinct antigenicity and their antigenic distances to their previous strains for each (sub)type of seasonal influenza viruses. Based on this relationship, we further develop a method to rapidly assess the mortality burden of influenza A(H1N1) virus by accurately predicting the antigenic distance between A(H1N1) strains. Rapid estimation of influenza mortality burden for new seasonal strains should help formulate a cost-effective response for influenza control and prevention.

## Author Summary

In epidemiology, investigators usually rely on surveillance data to assess the impact of an influenza virus on human health. However, accurate assessment of the influenza mortality burden at the early stage of influenza infection is rather challenging because the early influenza surveillance data are very limited and prone to bias as well. This speaks to an urgent need for the development of a more effective method for rapid and accurate estimation of influenza mortality burden. By proposing a novel host-virus interaction model, we have established a quantitative relationship between the antigenic variation of human influenza virus and its mortality burden. Based on this relationship, we further develop a method to rapidly assess the mortality burden of influenza A(H1N1) virus by accurately predicting the antigenic distance between A(H1N1) strains. We believe that our work will help develop a timely and sensible influenza preparedness programme that balances the gains of public health with the social and economic costs.

## Introduction

Seasonal influenza viruses have been and will continue to be a significant threat to public health [1]. There are three types of seasonal influenza – A, B and C. Among them, influenza H1N1 and H3N2 of type A and type B are currently circulating frequently in the human population. During evolution, many variants of influenza virus with distinct antigenic properties have emerged either by mutation or reassortment of the gene encoding the viral coat protein hemagglutinin (HA) [2]. These antigenic variants have caused morbidity and mortality with varying magnitude [3], [4]. If the mortality burden of a newly emerged virus can be estimated accurately and in time, this knowledge will be extremely valuable not only for public preparedness for an impending epidemic/pandemic but also for the health authorities to develop cost-effective control and intervention strategies.

In epidemiology, investigators usually rely on the surveillance data to assess the impact of an influenza virus on human death by estimating its case fatality ratio (CFR), the ratio of the number of deaths caused by the virus to the number of the diagnosed cases of the virus infection [5], and the excess mortality it causes [6], [7]. However, accurate prediction of the influenza mortality burden at the early stage of influenza infection is rather challenging, because the morbidity and mortality data for early influenza surveillance are very limited and prone to bias as well [5]. This speaks to an urgent need for the development of a more effective method for rapid and accurate estimation of the mortality burden of influenza virus.

It is generally assumed that the extent of an influenza virus to alter its antigenicity and to escape the pre-existing immunity in the human population determines its intensity of infection at the population level [8], [9] and thus the mortality burden. Despite the obvious causal relationship between the antigenic variations of influenza viruses and their mortality burdens, to our knowledge, no report has ever established a direct and positive correlation between them. In this study, based on the viral and mortality data of influenza in the USA and by using the excess all-cause mortality to represent the impact of influenza epidemics on human death [6], we describe our work on how to establish a positive correlation between the antigenic variation of human influenza virus and the total excess mortality it causes during all periods of its circulation. The established relationship has further enabled us to develop a method to rapidly estimate the mortality burden of influenza A(H1N1) virus by accurately predicting the antigenic distance between A(H1N1) strains based on their HA sequences.

## Results

### A simplified host-virus interaction model

**Figure 1:**
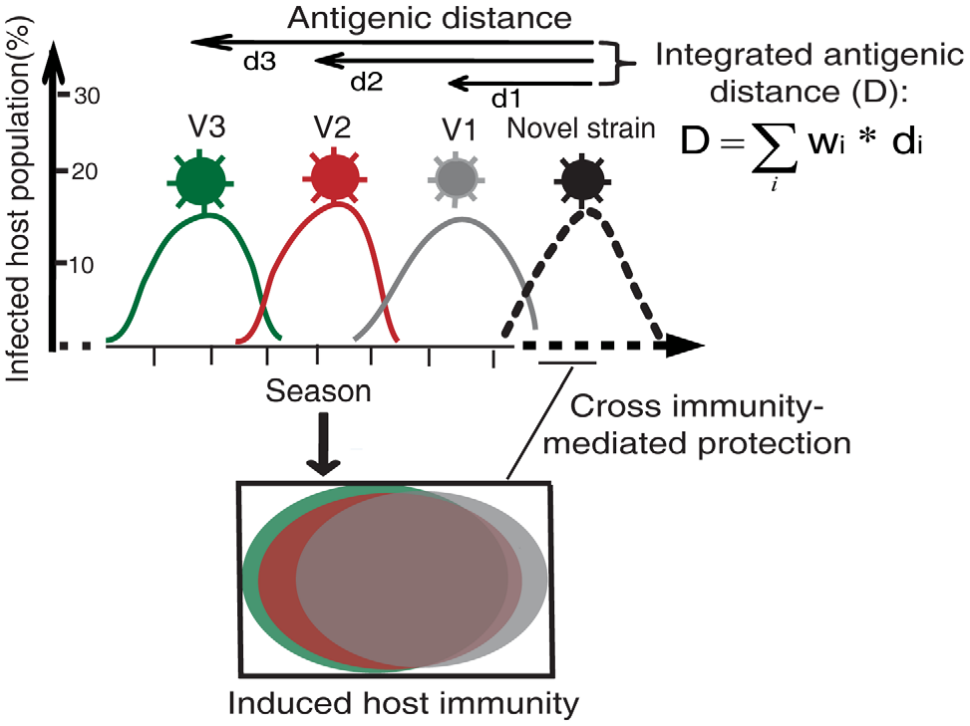
A simplified host-virus interaction model. The V1. V2, V3, … represent the first, second, third, … antigenic strain that circulated prior to a novel antigenic strain. To represent the ability of a novel strain to escape the pre-existing human immunity, we introduce a metric, integrated antigenic distance (D), as a linear combination of the antigenic distances between the novel virus and its previous antigenic strains (d).

To explore the relationship between antigenic variations of influenza viruses and the excess mortalities they cause, we proposed a simplified host-virus interaction model (Figure 1) by only considering the strains of distinct antigenicity that cause significant extent of infection in the human population. For convenience, these strains are denoted as “antigenic strains” (see Text S1 and Table S1 for details). In this model, only the strains of same influenza (sub)type are considered because there is little cross immunity between strains from different (sub)types [8]. Therefore, the evolution of a human influenza virus, such as A(H1N1), is considered as serial replacements of the antigenic strains driven by the cross immunity induced by the previous prevalent antigenic strains of same (sub)type in the human population (Figure 1). Given that the extent of cross protection between two viruses depends largely on the antigenic distance between them [8], it is likely that the antigenic distances between a new antigenic strain and the previous antigenic strains largely determine the mortality burden of the new strain on the human population.

### Correlation analysis of influenza virus excess mortality and antigenic variation

On the basis of the simplified host-virus interaction model, to establish correlation between the antigenic variations of an antigenic strain and the total excess mortality it may cause, we first looked into the contribution of a previous antigenic strain to induce pre-existing immunity and cross protect infection by a challenging strain. Table 1 shows the results of correlation analyses of influenza excess mortality and antigenic distance using Spearman and Pearson correlation methods [10] (see Materials and Methods for details). For A(H1N1) and A(H3N2) viruses, use of both methods shows there is a strong positive correlation (as reflected in Pearson's Correlation Coefficient (PCC) and Spearman's Correlation Coefficient (SCC)) between the total excess mortality caused by the challenging strain and its antigenic distance to the first antigenic strain (PCC = 0.79 (P-value = 0.03), SCC = 0.64 (P-value = 0.14) for A(H1N1); PCC = 0.58 (P-value = 0.03), SCC = 0.71 (P-value = 0.004) for A(H3N2)) and the second antigenic strain (PCC = 0.65 (P-value = 0.12), SCC = 0.64 (P-value = 0.14) for A(H1N1); PCC = 0.31 (P-value = 0.29), SCC = 0.21 (P-value = 0.46) for A(H3N2)). This indicates that the previous two antigenic strains, particularly the first one, produce the most protective immunity in the human population against the A(H1N1) and A(H3N2) epidemics.

**Table 1:** The Spearman and Pearson Correlation Coefficients between the excess all-cause mortalities and antigenic distances to previous individual antigenic strains.

Virus (sub)type	Correlation method	Previous antigenic strain^a^				
		1^st^	2^nd^	3^rd^	4^th^	5^th^
A(H1N1)	Spearman	**0.64(0.14)**	0.64(0.14)	−0.12(0.83)	-b	-
	Pearson	**0.79(0.03)**	0.65(0.12)	−0.25(0.63)	-	-
A(H3N2)	Spearman	**0.71(0.004)**	0.21(0.46)	−0.12(0.69)	0.13(0.65)	0.02(0.95)
	Pearson	**0.58(0.03)**	0.31(0.29)	−0.08(0.77)	0.19(0.53)	0.17(0.56)
B	Spearman	0.26(0.46)	0.57(0.11)	**0.74(0.045)**	0.07(0.88)	−0.58(0.23)
	Pearson	0.36(0.31)	0.53(0.14)	**0.84(0.009)**	0.14(0.77)	−0.5(0.31)

The numbers in parenthesis indicate the P-values of corresponding coefficients. The largest coefficient for each (sub)type is highlighted in bold.

a The previous *i* ^th^ antigenic strain is the *i*-th antigenic strain prior to an antigenic strain that is considered as a challenging strain.

b Not applicable due to the limited number of antigenic strains.

Interestingly, for type B virus, although there is a positive correlation between the total excess mortality caused by the challenging strain and its antigenic distances to the previous three antigenic strains, the best correlation is with the third antigenic strain (PCC = 0.84 (P-value = 0.009); SCC = 0.74 (P-value = 0.045)).

To further investigate the effect of previous antigenic strains combined on the excess mortality of a challenging strain, we first integrated the antigenic distances between the challenging strain and the previous antigenic strains of different numbers (see Text S1), then analyzed the correlations between the integrated antigenic distances and excess mortality using both Pearson and Spearman correlation methods. As shown in Table 2, for A(H3N2) and B viruses, there is no significant improvement on the correlations with the combined antigenic strains (compare Table 1 with Table 2, the best correlations in both tables and tests are highlighted in bold). While for A(H1N1) virus, the correlations with the combined antigenic strains are significantly improved in both Pearson and Spearman correlation tests. In Spearman test, the correlation coefficients increase from 0.64 (P-value = 0.14) to 0.75 (P-value = 0.066) when the two previous antigenic strains combined, and even to 0.94 (P-value = 0.017) when the previous three antigenic strains combined. Similarly, in Pearson test, the correlation coefficient increases from 0.79 (P-value = 0.03) to 0.91 (P-value = 0.005) when two previous antigenic strains combined. Taken together, we have not only discovered a significant correlation between the influenza virus excess mortality and its antigenic variation, but also established their best correlation for the three (sub)types of human viruses.

**Table 2:**
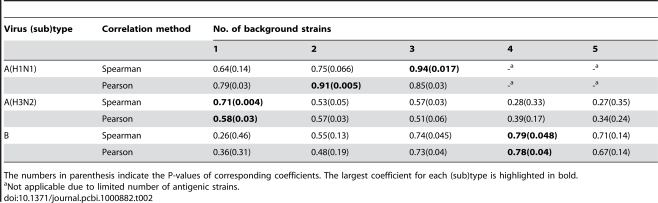
The Spearman and Pearson Correlation Coefficients between the excess all-cause mortalities and the integrated antigenic distances relative to the previous 1–5 antigenic strains as background strains.

The numbers in parenthesis indicate the P-values of corresponding coefficients. The largest coefficient for each (sub)type is highlighted in bold.

a Not applicable due to limited number of antigenic strains.

The genetic distance is another metric widely used to quantify the genetic variation between viruses. We further analyzed the correlation of influenza virus excess mortality and genetic distance for A(H1N1), A(H3N2) and B viruses (Table S2 and Table S3). Both Pearson and Spearman tests show that the correlation of excess mortality with genetic distance is not as significant as with antigenic distance (compare Table S2 to Table 1, and compare Table S3 to Table 2). These analyses demonstrate that antigenic variation rather than genetic variation is a good predictor for estimation of the excess mortality.

### Establishment of a quantitative relationship between excess mortality of A(H1N1) virus and its antigenic variation

The remarkable correlation between antigenic distance and excess mortality opens a new avenue to estimate the mortality burden of a novel antigenic variant that could potentially cause an influenza epidemic or pandemic. Here we sought to develop an approach to rapidly estimate the mortality burden of influenza A(H1N1) viruses, which is the most common cause of influenza (flu) in humans. To develop the approach, we first need to establish a quantitative relationship between the mortality burden of an A(H1N1) antigenic variant and its antigenic distances to previous antigenic strains, and then develop a computational model to predict antigenic distances between A(H1N1) viral strain based on their HA sequences.

**Figure 2:**
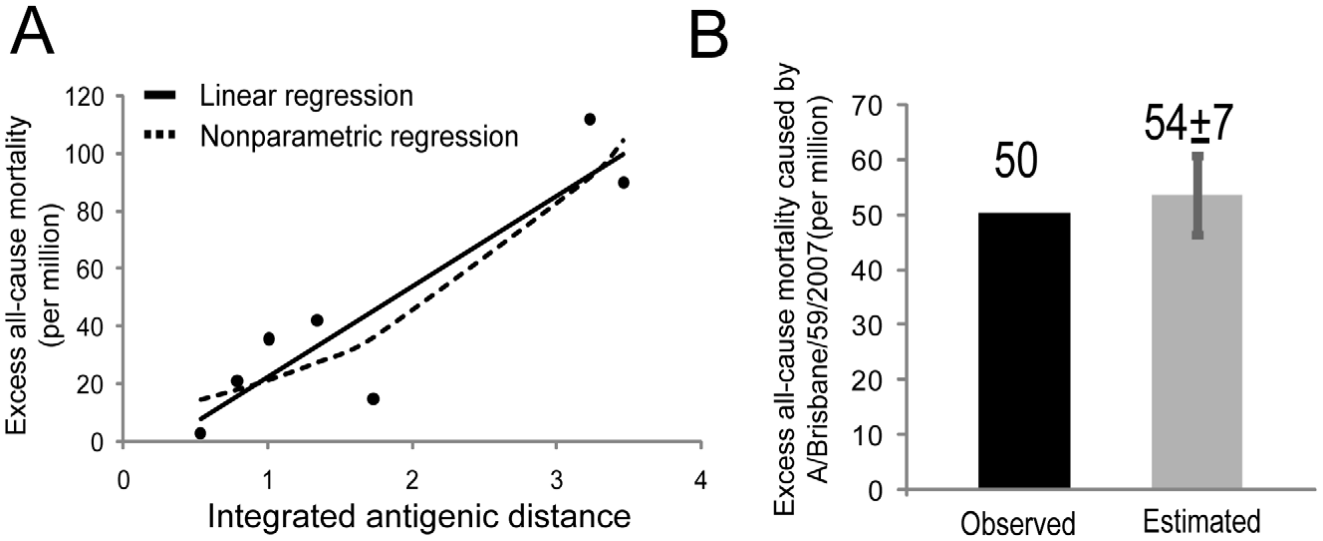
The quantitative relationship between the excess all-cause mortality and antigenic distance for A(H1N1). (A) The nonparametric (the dashed line) and ordinary linear (the black line) regression between the excess all-cause mortalities caused by A(H1N1) antigenic strains and their integrated antigenic distances to the previous first and second antigenic strains. The nonparametric regression is done using the local polynomials method (called loess method [11]). (B) The observed and estimated excess all-cause mortality for recent seasonal A(H1N1) virus A/Brisbane/59/2007. The error bar shows the standard deviation of the prediction.

To establish a quantitative relationship between the mortality burden and antigenic variation for A(H1N1) virus, we considered the integrated antigenic distance between a challenging strain and the previous two antigenic strains because the Pearson test gave the best correlation with statistical significance for the previous two antigenic strains. Figure 2A show the fittings of a non-parametric local polynomials model and a parametric linear regression model to the integrated antigenic distance (D) and the excess mortality (M). Both models demonstrated a monotonic increase of the excess mortality with the increase of the integrated antigenic distance, reinforcing a positive correlation between them. For simplicity, we used a traditional least squares linear regression model to describe their quantitative relationship (Figure 2A), which is described by the following equation:
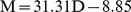
(1)The standard deviations of D and constant terms are 6.42 and 13.11, respectively. The linear fit yields a PCC of 0.91 (P-value = 0.005), indicating a high accuracy of using the equation to predict the excess mortality from the integrated antigenic distance. To assess whether the above linear relationship is robust to outliers, we carried out robust regression that limits the influence of outliers (Table S4). The robust regression shows a very stable linear relationship between excess mortality and integrated antigenic distance (P-value = 2.51e-05), which is very similar to that given by the traditional least squares linear model. To further assess the quality of the Equation 1 in prediction, we carried out leave-one-out cross validation (Figure S1). The leave-one-out test shows that the predicted excess mortalities highly correlate with the observed excess mortalities (PCC = 0.80), indicating the reliability of using the linear model to quantify the relationships for the data points we have.

The recent seasonal A(H1N1) virus A/Brisbane/59/2007, which started to circulate in humans since the 2007–2008 season, has caused excess mortality of 50 per million as of the 2008–2009 season (Figure 2B), and is unlikely to cause significant deaths due to its very low infections since May 17, 2009 [12]. Since A/Brisbane/59/2007 is not included in the construction of our model, the application of the Equation 1 to estimate its excess mortality would provide a blind test for our model. To estimate its total excess mortality, we computed the integrated antigenic distance between the A/Brisbane/59/2007 virus and its previous two antigenic strains, A/New Caledonia/20/1999 and A/Solomon Islands/3/2006 (Table S1), and then used Equation 1 to estimate the total excess mortality caused by A/Brisbane/59/2007. The estimated total excess mortality is 54 per million, close to the actual number of deaths it caused (Figure 2B), indicating the validity of our method in predicting excess mortality of seasonal A(H1N1) virus.

### Development of a computational approach to rapidly quantify the antigenic distances among different strains of human influenza A(H1N1) virus

**Figure 3:**
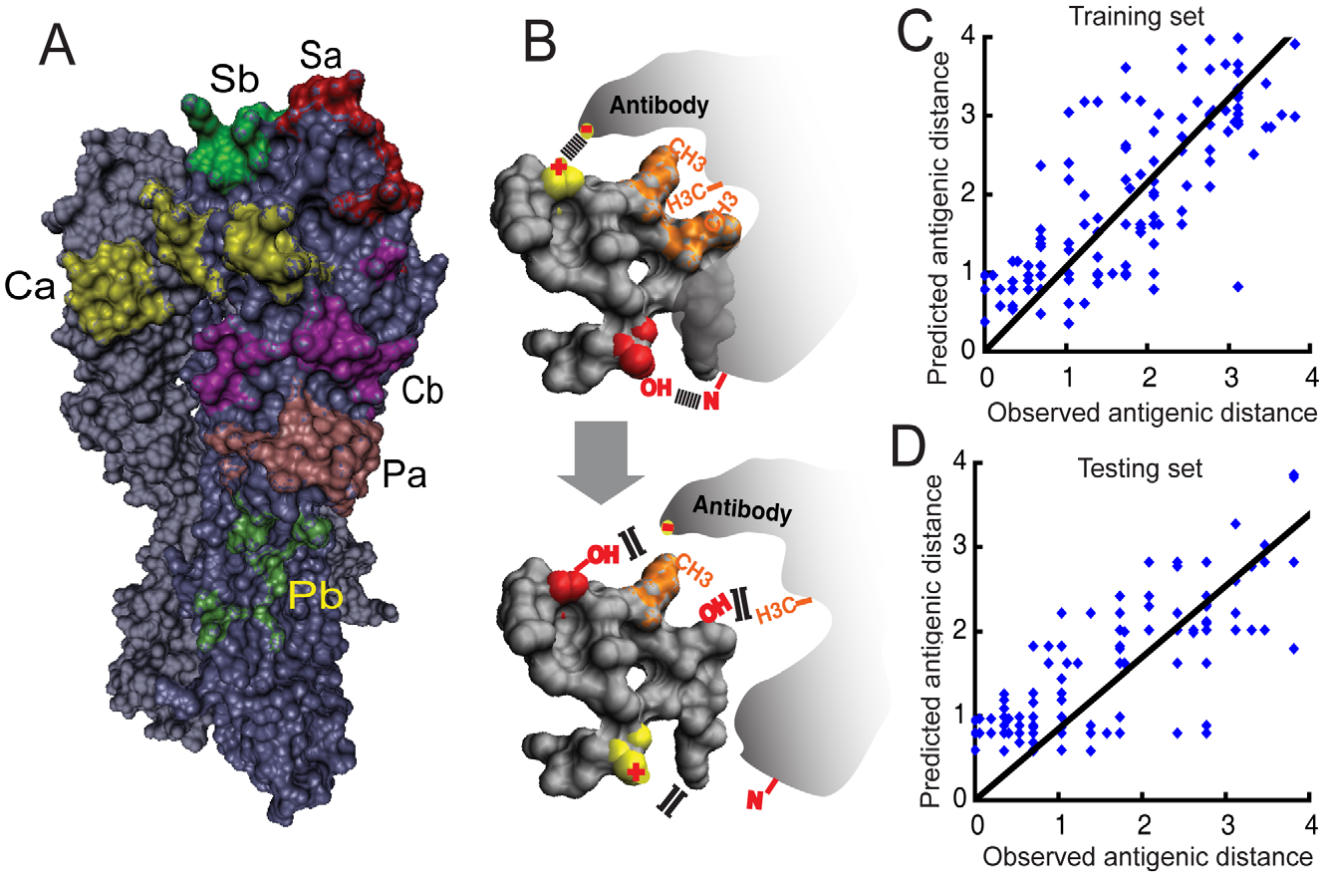
The epitope-based antigenic distance prediction (EADpred) model and its performance. (A) Six derived antigenic epitopes (Sa, Sb, Ca, Cb, Pa and Pb) on human influenza A(H1N1) HA protein were considered as the structural basis underlying the interactions between HA and neutralizing antibodies. They were marked on the surface of the structure model of HA of A/putertorico/8/34 (H1N1) virus (PDB ID: 1RVZ) using different colors. (B) A cartoon illustrating the physicochemical mechanisms underlying an epitope-mediated interaction between HA and antibody. Salt-bridge interaction was shown by a link between two charged atoms. Hydrogen bonding was represented as a link of “-OH—N-”. Hydrophobic microenvironment was described as a cluster of hydrophobic groups highlighted in orange. (C–D) The prediction performances on the training data (C) and testing data (D). Black lines reflect linear fit with a zero intercept. The linear fits to the training data and testing data yield correlation coefficients of 0.79 and 0.80 respectively. The details for the model description see the Materials and Methods.

To develop a rapid tool to predict the excess mortality to be caused by a novel A(H1N1) antigenic variant, we need to determine its antigenic distances to its previous two antigenic strains. However, determining the antigenic distance between viruses using experiments such as hemagglutination inhibition (HI) assay is time-consuming and labor-intensive [13]. The problem is more severe when determining the antigenic distances for a novel strain, because it may take up to month to obtain the antisera against the novel strain. Therefore, we are interested in developing a computational model to predict the antigenic distances between viral strains based on their HA sequences. Although many studies have attempted to identify the antigenic variants of human influenza viruses based on the amino acid changes on HA [14], [15], the computational method to quantify the antigenic changes of influenza A(H1N1) virus is not available. To this end, we developed a novel computational approach, Epitope-based Antigenic Distance Prediction (EADpred), to estimate the antigenic distance between A(H1N1) strains with high speed and accuracy (Figure 3A, B). Details of the development of the method were described in the Materials and Methods. Figure 3C shows the correlation between the predicted antigenic distances and the observed antigenic distances over the training data: the overall PCC is 0.79 (Table S5). Figure 3D shows that the EADpred also does well on a testing data set consisting of 172 experimentally determined antigenic distances between A(H1N1) strains of known HA sequences (PCC = 0.80) (Table S5). The ability of EADpred to predict antigenic distance with a relatively high accuracy suggests its potential utility in the rapid estimation of influenza mortality burden.

### Predicted antigenic variation of A(H1N1) virus correlates with its total excess mortality

**Figure 4:**
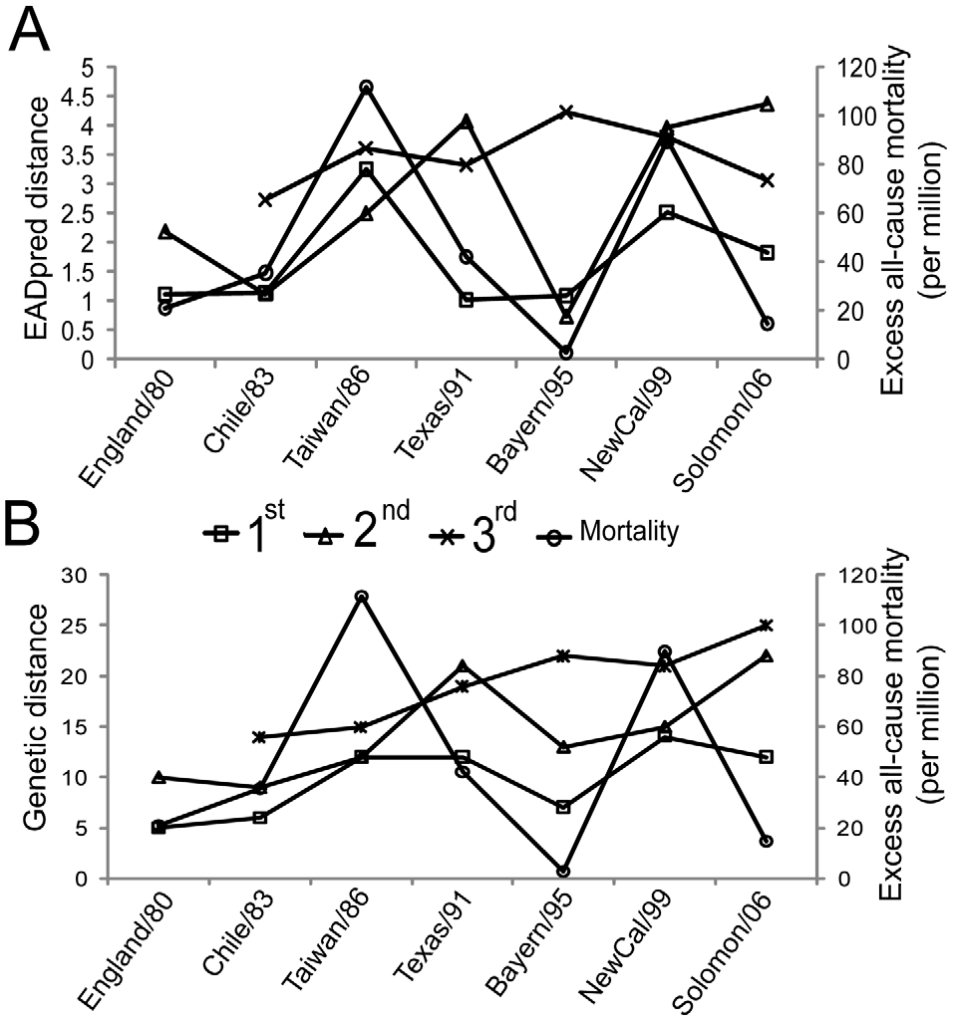
Correlation of predicted antigenic distance (EADpred distance) with excess mortality caused by A(H1N1). (A–B) The correlation between excess all-cause mortality (black) and EADpred (A) /genetic (B) distance to previous single antigenic strains. The symbols Ο, □, Δ and × indicate the excess all-cause mortality and the antigenic/genetic distances between antigenic strains as challenging strains and the previous first, second, and third antigenic strains, respectively. Information about the antigenic strains sees Table S1.

We further evaluated how well the predicted antigenic distance, also called EADpred distance, of A(H1N1) virus correlates with the observed excess mortality. We carried out the same correlation analyses as we did for the observed antigenic distance described above by substituting the observed antigenic distances with the EADpred distances (Figure 4A). Indeed, the EADpred distance correlates strongly with the excess mortality (PCC = 0.86, P-value = 0.01). Although the correlation is slightly weaker as compared to that of the observed antigenic variation (PCC = 0.91, P-value = 0.005), it is much stronger than that of the genetic variation (PCC = 0.61, P-value = 0.15), which is not significant (Figure 4B). Nowadays the determination of HA sequence of influenza virus has been become a routine work in influenza surveillance due to the availability of rapid, inexpensive and high-throughput sequencing technology, the development of sequence-based computational method for reliably predicting antigenic distance will enable an estimation as early as possible of excess mortalities for emerging antigenic strains.

## Discussion

In this study, by proposing a simplified virus-host interaction model, we have discovered a direct and positive correlation between the extent of antigenic variation of an influenza virus and the total excess mortality it may cause. The impact of influenza on human death has been a long-standing focus of influenza studies. Many factors have been thought to contribute to influenza mortality burden including viral factors such as the pathogenicity-related molecular markers [16], receptor binding [17] and antigenic variation of the influenza virus [8], as well as many external factors such as age structure of the human population, effectiveness of influenza vaccine, temperature and humidity [18]–[20]. Despite these many potential contributing factors, we found a significant correlation between the extent of antigenic variation of an influenza virus and its impact on total excess mortality. This relationship is valid for all three human influenza viruses analyzed: A(H1N1), A(H3N2) and B. Recently, Park et al [21] also found a positive correlation between the probability of being infectious and the number of amino acid differences in antigenic sites of HA based on their experiments on equine influenza virus. Therefore we argue that for an influenza virus that has been adapted in its host, the antigenic variation is the most important factor that determines its mortality burden. Although somewhat surprising, this is reasonable because the human-adapted influenza viruses like seasonal viruses generally have considerable changes only in antigenicity but not in the pathogenicity and receptor specificity.

A major challenge in correlation analysis of the antigenic variation of influenza virus and its mortality burden is to attribute the excess mortality to a specific influenza strain. Although the excess mortality attributed to all influenza viruses in a given period of time can be inferred with high confidence from the mortality data reported [6], [7], further attribution of the excess mortality to a specific strain based on current limited surveillance data is very difficult. In this study, we estimated the excess mortality caused by a virus based on its virus isolation rate, the proportion of clinical samples positive that was reported for the virus in the given period of time (see Materials and Methods in detail). The virus isolation rate from clinics reflects the virus's ability to infect and its ability to cause disease as well because people with disease are more likely to go to clinic for viral testing than those without. For the same (sub)type of virus, the ability to cause disease may reflect the ability to cause death. Figure S2 shows that the combined three (sub)type-associated excess mortality inferred based on virus isolation rate correlates significantly with the influenza-associated excess mortality calculated from reported mortality data. This suggests that the viral isolation rate could largely reflect the virus's ability to cause death. But we also observed several large deviations between the predicted total excess mortality and reported total excess mortality (see the boxed dots in Figure S2). These deviations could be not only due to data noise and but also due to different ability of different viruses to cause death. It is worthy of being noted that, in case of the emergence of a particularly severe strain, our calculation could lead to a significant underestimation of the excess mortality caused by the virus. Therefore, our calculated excess mortality based on the virus isolation rate may underestimate the magnitude of differences in excess mortality between viruses.

Although the correlation between the antigenic variation of influenza virus and its mortality burden is impressive, a major limitation of our work is that most of the correlation coefficients have large confidence intervals (see Table S6 and Table S7), leading to the relatively high standard deviation of the coefficient in the regression model (see Equation 1) and the large confidence interval of the prediction for A/Brisbane/59/2007 ([3, 104] on 95% confidence level). This may be due to the data noise and the limited data available. Therefore, we think that our results need to be further validated once data from additional seasons available.

Human influenza A viruses will continue to have significantly negative impact on public health and cause substantial morbidity and mortality. Timely and accurate estimation of their impact on human death will help formulate more sensible and cost-effective influenza prevention and control policies. The discovery of a significantly positive correlation between antigenic variation of an influenza virus and its excess mortality has allowed us to further establish a quantitative relationship between them. In addition to A(H1N1), we also quantified the relationship between antigenic variation and excess mortality for influenza A(H3N2) and B virus (see Figure S3 and Table S8 for A(H3N2), and Figure S4 and Table S9 for B). The established quantitative relationship for a (sub)type could be applicable to any challenging antigenic strain of same (sub)type, if its predecessors could be known that provided cross-protection for it and caused a wide infection in the current population. In the case of 2009 Swine-origin Influenza A(H1N1) Virus (S-OIV), the direct application of the established relationship is not appropriate as its predecessors provided little cross-protection except for a marginal cross-protection observed in some people aged over 65 [22]. Given its antigenic variation much larger than seasonal A(H1N1) antigenic strains, we speculate that the 2009 S-OIV would cause a larger mortality than past seasonal A(H1N1) viruses.

Since the current experimental methods in determining antigenic distances between viral isolates are time-consuming, we further proposed a sequence-based approach, EADpred, to predict antigenic distance between A(H1N1) viral strains, which enables us to rapidly assess the mortality burden of an A(H1N1) antigenic variant. Our method only relies on the HA sequence data of the influenza viruses rather than the surveillance data, which offers a rapid and reliable tool to assess the potential impact of an influenza virus on human death even before infection occurs in humans. Since a rapid and accurate prediction of influenza mortality burden should greatly help develop a timely and sensible preparedness programme that balances the gains of public health and the social and economic costs, we believe that our method will be very useful for rapid assessment of the influenza mortality burden of other future A(H1N1) variants, and is also applicable to the antigenic variants of human A (H3N2) and B viruses with proper modification.

## Materials and Methods

### Data sources

HI data, HA sequences, the US mortality data, the US population data and other surveillance data regarding influenza in the US including number of total respiratory specimens tested for influenza and positive isolates of three human influenza viruses, A(H1N1), A(H3N2) and B from season 1977–1978 through 2008–2009 were collected from published records, documents or databases (see ). The antigenic strains for each (sub)type of human influenza virus were defined based on the vaccine strains recommended by World Health Organization (WHO) or the reference strains used by US Centers for Disease Control and Prevention (CDC) in influenza surveillance. These strains were selected from the US CDC reports or related documents, which were required to be dominant (comprising >50% of the total isolates of the same (sub)type) in at least one flu season based on the influenza surveillance by the US CDC. Their actual circulation time was based on the influenza surveillance carried out by the US CDC. See Table S1 for the detailed information regarding the antigenic strains. See Text S1 for detailed information about these data sources and data processing.

### Antigenic distance

Antigenic distance between two strains *i* and *j*, *d_ij_*, was calculated based on HI data by following the Archetti-Horsfall method with adaption [23], [24]:
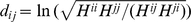
(2)where H^ij^ refers to the HI titer of *i* relative to antisera raised against *j*. In developing the EADpred model, only the antigenic distances between strains of human A(H1N1) virus with HA1 peptide sequences (1–331 resides) available were considered. The training dataset consists of 143 antigenic distances between A(H1N1) strains, in which at least one strain in each pair was isolated between 1977 and 1999. The testing dataset consists of 172 antigenic distances between A(H1N1) strains that were both isolated between 2000 and 2008 (Table S10).

### Estimation of total excess all-cause mortalities attributed to antigenic strains

The excess all-cause mortalities for seasons 1977–1978 through 2008–2009 were calculated by following Simonsen's method [6]. In our study, the estimation of excess mortality attributed to an antigenic strain in a given period of time is based on the virus isolation rate, the proportion of clinical samples that was tested positive for the antigenic strain in the given period of time. The virus isolation rate for a (sub)type in a given season is calculated as the number of virus tested positive for the (sub)type divided by the total number of clinical samples tested in the season. To link excess mortality to virus isolation rate for a (sub)type, we introduce a metric Y, which is defined as the excess mortality per UNIT of virus isolation rate. Therefore, the estimation of the total excess all-cause mortality attributed to an antigenic strain consists of the following three steps:

*Step 1*: Determination of Y for each (sub)type. To determine the Y for a (sub)type, we first identified the seasons in which the (sub)type dominates almost completely (the ratio of virus isolates of the (sub)type exceeding 90 percent of the total isolates in the season). Then the Y for the subtype is calculated over the identified dominant seasons using the following formula:
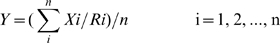
(3)where Xi is the excess all-cause mortality in the dominant seasons, Ri is the proportions of samples positive for the (sub)type, and n is the number dominant seasons.

*Step 2*: Calculation of the excess all-cause deaths associated with a (sub)type for each season from 1977–1978 through 2008–2009. The excess mortality in a season was obtained by multiplying by Y the proportions of samples positive for the (sub)type in the season.

*Step 3*: Calculation of the total excess all-cause mortality for an antigenic strain. For each antigenic strain in a given (sub)type, its total excess all-cause mortality is calculated as the sum of the excess (sub)type-attributed all-cause mortalities in the flu seasons when it was in circulation. For a season with over one antigenic strains of the same (sub)type in circulation, the excess all-cause mortality caused by an antigenic strain is estimated based on the percentage of its antigenically similar isolates in the same (sub)type characterized by the US CDC in the given season (Table S1).

### Correlation analysis of antigenic distance and excess all-cause mortality

For the correlation analysis, we only considered the antigenic strains that have completed the whole circulation (from the beginning of circulation to the end of circulation) from 1977 through 2009 (Table S1). The correlation analysis was carried out, respectively, for all three (sub)types of human influenza viruses, A(H1N1), A(H3N2) and B, in the flu seasons from 1977–1978 through 2008–2009. For each (sub)type, the correlation analysis was performed as follows. First, the antigenic distances between an antigenic strain and its previous antigenic strains were obtained from Table S10. Then, the correlations between the total excess all-cause mortalities caused by antigenic strains and their antigenic distances to the previous *i*-th antigenic strain were analyzed using the PCC and SCC in the R package [10]. Finally, the antigenic distances to the previous two antigenic strains were combined to obtain a better correlation between the antigenic variation of an antigenic strain and the total excess mortality it causes (The calculation of integrated antigenic distance sees Text S1). For comparison, the correlation between genetic distance (number of amino acid differences on HA1 peptide) and excess all-cause mortality was also computed following the same procedure described above.

### Epitope-based Antigenic Distance Prediction (EADpred)

The development of the EADpred consists of four steps described in brief as follows (details see Text S1):

*Step 1*. Identification of the antigenic epitopes as structural base underlying the HA-antibody interaction.

We have derived six antigenic epitopes in the HA of A(H1N1) virus, including four expanded known antigenic epitopes (Sa, Sb, Ca and Cb) and two novel antigenic epitopes (Pa and Pb) (Figure 3A). The composition of the six antigenic epitopes and the supporting sources are summarized in Table S11.

*Step 2*. Transformation of amino acid changes in an antigenic epitope into the changes in physicochemical properties that underlie HA-antibody interaction.

The amino acid changes in an antigenic epitope were transformed into a linear combination of physiochemical proterties as follows:
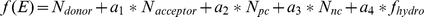
(4)where f(E) quantifies the changes in the physiochemical properties in the antigenic epitope. N_donor_ and N_acceptor_ represent the number of the changed hydrogen-bond donors and acceptors. N_pc_ and N_nc_ represent the number of the positive changes and negative charges. 
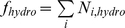
 represents the changed hydrophobicity of the epitope that consists of several cavities with at least two amino acids, which is a sum of hydrophobicities of these cavities. All these physiochemical properties of the 20 amino acids were assigned to a vector of values as listed in Table S12.

*Step 3*. Integration of the contributions of the six derived antigenic epitopes to predict the antigenic distance.

To predict the antigenic distances between two viral strains (d), we considered a linear combination of the changes in physicochemical properties in all the six derived antigenic epitopes:
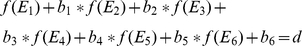
(5)where f(E_i_) denotes the function of the *i*-th epitope.

Then the Equation 4 and 5 were combined into one equation, which is re-represented as follows:
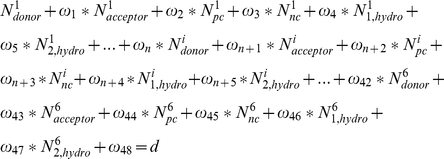
(6)where 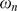 are relative weights of the different terms. N^i^_donor_, N^i^_acceptor_, N^i^_pc_ and N^i^_nc_ are the four variables describing the effects of the hydrogen bonding and salt bridge of the *i*-th epitope that was described in Equation 5. 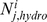 is the *j*-th cavity of the *i*-th epitope.

*Step 4*. Model parameterization and assessment.

The relative weights of the Equation 6 were parameterized on the training dataset using a stepwise multiple regression. After regression, we found a certain linear correlation between antigenic distance and the number of terms with non-zero weight. Therefore, to achieve a better prediction performance, we added in our previous model another term, 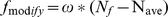 where 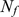 is the number of terms with non-zero weight left after linear regression of Equation 6, 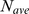 is the averaged 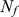 over all strain pairs in the training dataset, 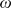 is the weight of the term. The prediction performance of the model parameterized on the training was further assessed by a blind test in the testing set. The testing results were shown in Figure 3D and Table S5. The performance of our method in predicting antigenic variants is also compared to one of the best previous site-based methods (see Text S1 and Table S5).

### Statistical analysis in this study

In this study, all statistical analyses including the use of Spearman and Pearson correlation methods were carried out using the statistical package R [10]. The correlation analysis was done with the cor.test function. The classical, robust and nonparametric regression is done with the lm, lmRob and loess function respectively.

## Supporting Information

Figure S1 The leave-one-out cross validation of the linear regression for analyzing the relationship between the excess all-cause mortality caused by an antigenic strain and its integrated antigenic distance to its previous two strains. Each time, the excess all-cause mortality caused by an antigenic strain and its integrated antigenic distance to its previous two strains were removed and a linear equation was fitted to the remaining data. Using the fitted equation, we then predicted the total excess mortality caused by the antigenic strain based on its integrated antigenic distance to its previous two antigenic strains.
(0.34 MB TIF)

Figure S2 The scatterplot of the sum of (sub)type-attributed excess mortality that we calculated and the reported excess mortality in each season. The red, blue and green points represent the A(H1N1), A(H3N2) and B dominant seasons respectively. A (sub)type is defined to be dominant in the season when its ratio of virus isolates is the biggest in that season. The black line is the diagonal line of the plot. The boxed dots are those with large deviations.
(0.42 MB TIF)

Figure S3 The nonparametric (the red line) and robust logarithm (the black line) regression between the excess all-cause mortality and the antigenic distance to the previous first antigenic strain for influenza A(H3N2) virus. The nonparametric regression is done using the loess method with span 1.5. The equation and its R-squared shown on the plot are for the robust logarithm regression.
(0.34 MB TIF)

Figure S4 The nonparametric (the red line) and robust logarithm(the black line) regression between the excess all-cause mortality and the antigenic distance to the previous third antigenic strain for influenza B virus. The nonparametric regression is done using the loess method with span 1.5. The equation and its R-squared shown on the plot are for the robust logarithm regression.
(0.33 MB TIF)

Table S1 The seasonally virus isolates, antigenic strains and excess all-cause mortalities of human influenza A(H1N1), A(H3N2) and B from the year 1977 through 2009.
(1.07 MB TIF)

Table S2 The Spearman and Pearson Correlation Coefficients between the excess all-cause mortalities and the genetic distances to its previous individual antigenic strains. The numbers in parenthesis are the P-values of the corresponding coefficients. The largest coefficient for each (sub)type is highlighted in bold. a: The previous i-th antigenic strain is the i-th antigenic strain prior to an antigenic strain that is considered as a challenging strain. b: Not applicable due to the limited number of antigenic strains.
(0.03 MB DOC)

Table S3 The Spearman and Pearson Correlation Coefficients between the excess all-cause mortalities and the integrated genetic distances relative to the previous 1–5 antigenic strains as background strains. The numbers in parenthesis are the P-values of the corresponding coefficients. The largest coefficient for each (sub)type is highlighted in bold. a: Not applicable due to limited number of antigenic strains.
(0.03 MB DOC)

Table S4 The classical and robust regression analysis of the relationship between the antigenic distance and the excess mortality for human A(H1N1) using five different equations. The table lists the function, R-squared and P-value for each regression.
(0.04 MB DOC)

Table S5 The performance comparison between the EADpred method and one of the best site-based methods in predicting antigenic variants (see Methods). a: Based on the method proposed by Liao et al [11].
(0.03 MB DOC)

Table S6 The confidence interval of the Spearman and Pearson Correlation Coefficients between the excess all-cause mortalities and antigenic distances to previous individual antigenic strains. The numbers in parenthesis are the 95% confidence interval of corresponding coefficients. The numbers in red are the coefficients with P-value smaller than 0.05. a: The previous i-th antigenic strain is the i-th antigenic strain prior to an antigenic strain that is considered as a challenging strain. b: Not applicable due to the limited number of antigenic strains.
(0.03 MB DOC)

Table S7 The confidence interval of the Spearman and Pearson Correlation Coefficients between the excess all-cause mortalities and the integrated antigenic distances relative to the previous 1–5 antigenic strains as background strains. The numbers in parenthesis are the 95% confidence interval of corresponding coefficients. The numbers in red are the coefficients with P-value smaller than 0.05. a: Not applicable due to limited number of antigenic strains.
(0.03 MB DOC)

Table S8 The classical and robust regression analysis of the relationship between the antigenic distance and the excess mortality for human A(H3N2) using five different equations. The table lists the function, R-squared and P-value for each regression.
(0.04 MB DOC)

Table S9 The classical and robust regression analysis of the relationship between the antigenic distance and the excess mortality for human B virus using five different equations. The table lists the function, R-squared and P-value for each regression.
(0.04 MB DOC)

Table S10 Antigenic distances between antigenic strains for human influenza A(H1N1), A(H3N2) and B, and antigenic distances between A(H1N1) viruses used for developing the EADpred method.
(0.54 MB DOC)

Table S11 Six predicted epitopes of the A(H1N1) HA protein. a: The epitopes are extended from the known epitopes based on references 12–14. b: Two predicted novel antigenic eptiopes supported by references 17 and 18.
(0.03 MB DOC)

Table S12 Values of five selected physiochemical properties of the 20 amino acids. a: The hydrophobic values came from the BLAS910101 entry in AAindex database [21].
(0.05 MB DOC)

Text S1 Supporting methods, legends for supporting tables and figures.
(0.10 MB DOC)
