# Supplementary figures and images for: Correlation of Influenza Virus Excess Mortality with Antigenic Variation: Application to Rapid Estimation of Influenza Mortality Burden
Source: PLoS Comput Biol. 2010 Aug 12;6(8):e1000882. doi: 10.1371/journal.pcbi.1000882 (PMC2920844; doi:10.1371/journal.pcbi.1000882)

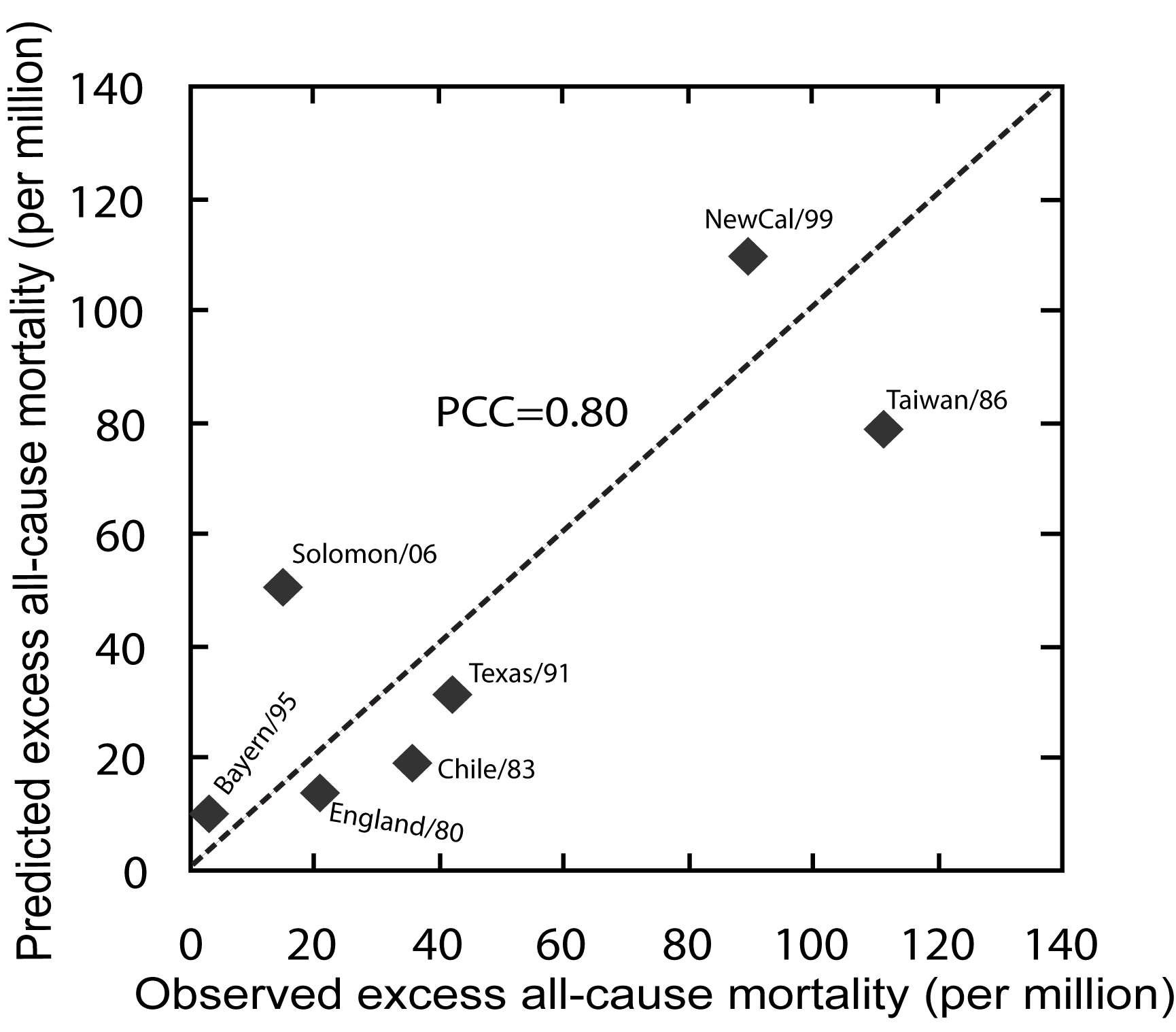

Supplement: Figure S1 — The leave-one-out cross validation of the linear regression for analyzing the relationship between the excess all-cause mortality caused by an antigenic strain and its integrated antigenic distance to its previous two strains. Each time, the excess all-cause mortality caused by an antigenic strain and its integrated antigenic distance to its previous two strains were removed and a linear equation was fitted to the remaining data. Using the fitted equation, we then predicted the total excess mortality caused by the antigenic strain based on its integrated antigenic distance to its previous two antigenic strains. (0.34 MB TIF) [file pcbi.1000882.s001.tif]

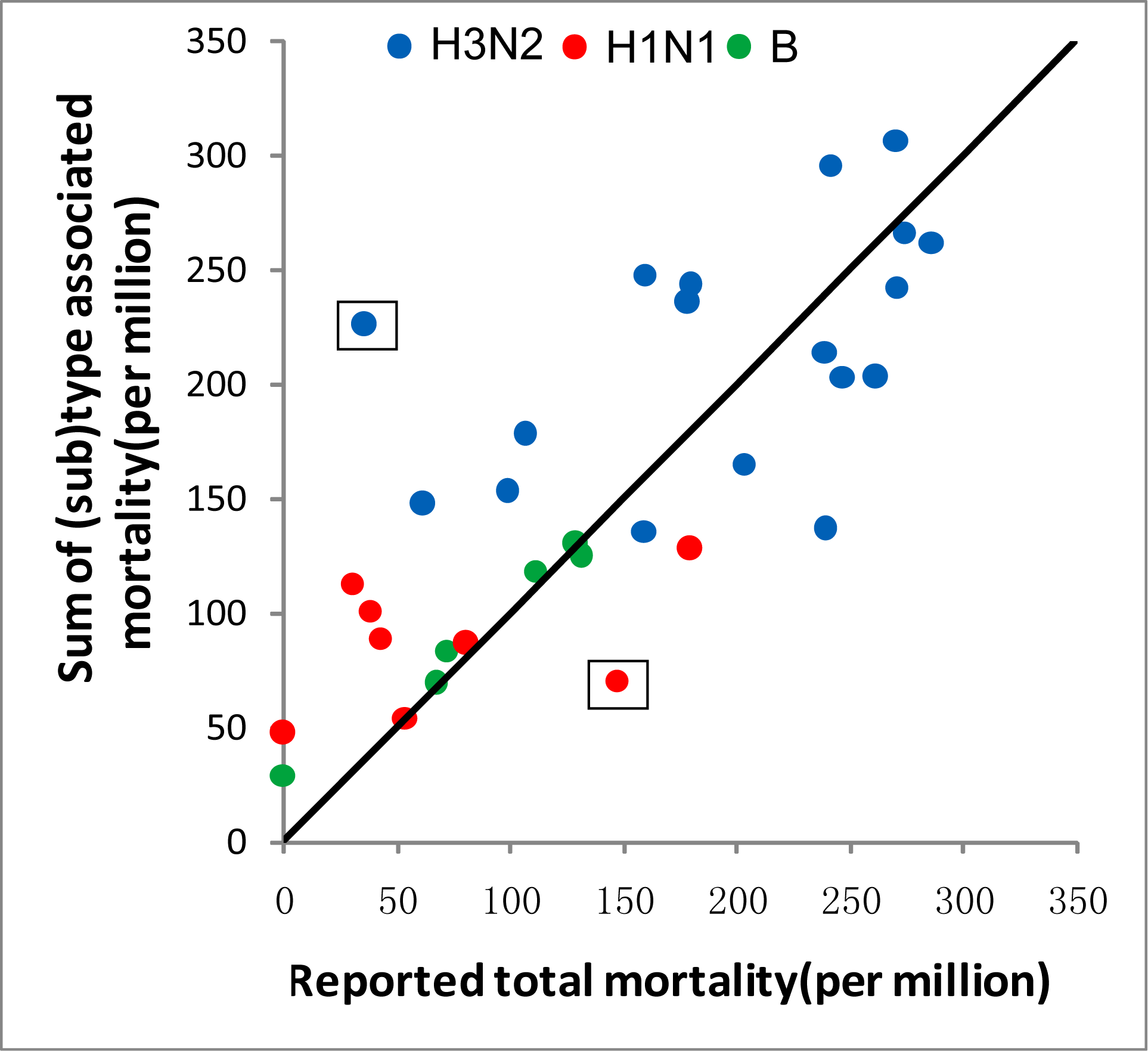

Supplement: Figure S2 — The scatterplot of the sum of (sub)type-attributed excess mortality that we calculated and the reported excess mortality in each season. The red, blue and green points represent the A(H1N1), A(H3N2) and B dominant seasons respectively. A (sub)type is defined to be dominant in the season when its ratio of virus isolates is the biggest in that season. The black line is the diagonal line of the plot. The boxed dots are those with large deviations. (0.42 MB TIF) [file pcbi.1000882.s002.tif]

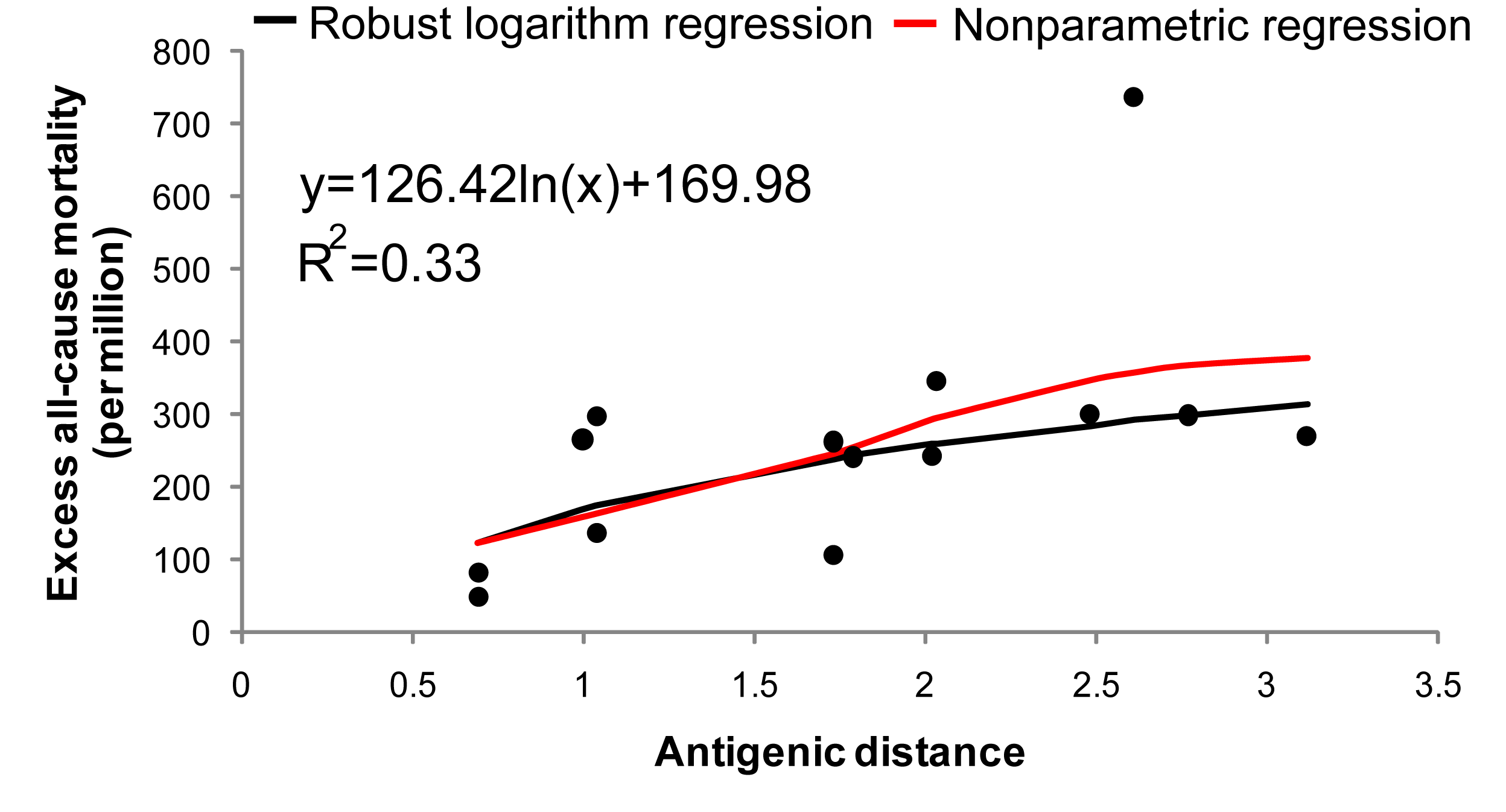

Supplement: Figure S3 — The nonparametric (the red line) and robust logarithm (the black line) regression between the excess all-cause mortality and the antigenic distance to the previous first antigenic strain for influenza A(H3N2) virus. The nonparametric regression is done using the loess method with span 1.5. The equation and its R-squared shown on the plot are for the robust logarithm regression. (0.34 MB TIF) [file pcbi.1000882.s003.tif]

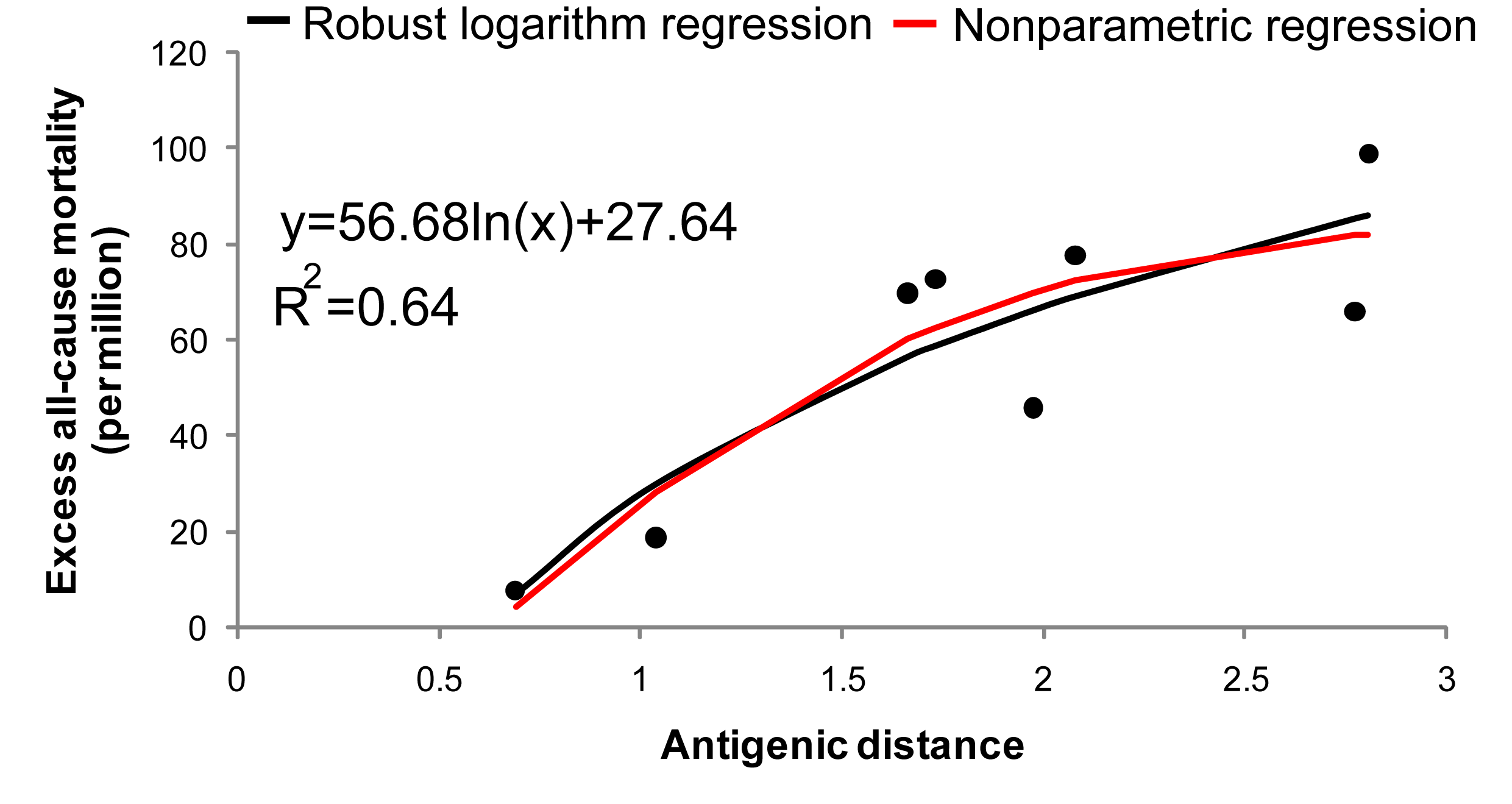

Supplement: Figure S4 — The nonparametric (the red line) and robust logarithm(the black line) regression between the excess all-cause mortality and the antigenic distance to the previous third antigenic strain for influenza B virus. The nonparametric regression is done using the loess method with span 1.5. The equation and its R-squared shown on the plot are for the robust logarithm regression. (0.33 MB TIF) [file pcbi.1000882.s004.tif]

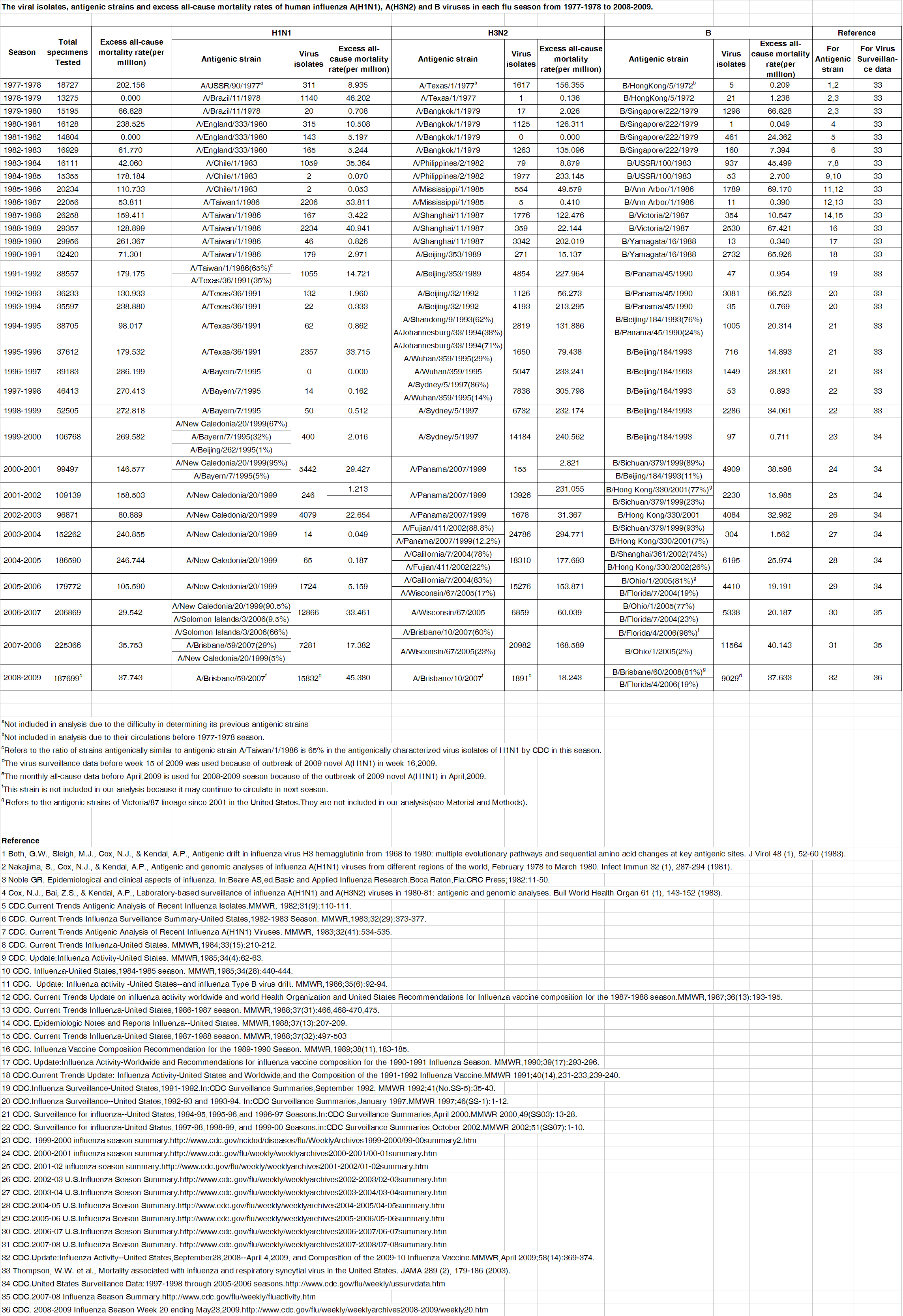

Supplement: Table S1 — The seasonally virus isolates, antigenic strains and excess all-cause mortalities of human influenza A(H1N1), A(H3N2) and B from the year 1977 through 2009. (1.07 MB TIF) [file pcbi.1000882.s005.tif]
