# Supplementary material for: Correlation of Influenza Virus Excess Mortality with Antigenic Variation: Application to Rapid Estimation of Influenza Mortality Burden
Source: PLoS Comput Biol. 2010 Aug 12;6(8):e1000882. doi: 10.1371/journal.pcbi.1000882 (PMC2920844; doi:10.1371/journal.pcbi.1000882)
Supplement: Table S2 — The Spearman and Pearson Correlation Coefficients between the excess all-cause mortalities and the genetic distances to its previous individual antigenic strains. The numbers in parenthesis are the P-values of the corresponding coefficients. The largest coefficient for each (sub)type is highlighted in bold. a: The previous i-th antigenic strain is the i-th antigenic strain prior to an antigenic strain that is considered as a challenging strain. b: Not applicable due to the limited number of antigenic strains. (0.03 MB DOC) [file pcbi.1000882.s006.doc]

| **Virus (sub)type** | **Variation** | **Previous antigenic strain^a^** | | | | |
| --- | --- | --- | --- | --- | --- | --- |
|  |  | **1^st^** | **2^nd^** | **3^rd^** | **4^th^** | **5^th^** |
| A(H1N1) | Spearman | **0.52(0.23)** | -0.11(0.84) | -0.54(0.3) | -^b^ | - |
|  | Pearson | **0.61(0.15)** | -0.13(0.77) | -0.49(0.32) | - | - |
| A(H3N2) | Spearman | 0.51(0.06) | **0.57(0.03)** | 0.1(0.73) | -0.09(0.76) | -0.08(0.77) |
|  | Pearson | 0.42(0.13) | **0.6(0.02)** | 0.19(0.52) | -0.12(0.68) | -0.22(0.44) |
| B | Spearman | -0.02(0.95) | -0.12(0.76) | **0.46(0.26)** | 0.07(0.88) | -0.12(0.82) |
|  | Pearson | -0.08(0.84) | 0.02(0.97) | **0.60(0.12)** | 0.29(0.53) | -0.22(0.68) |
