# Supplementary material for: Correlation of Influenza Virus Excess Mortality with Antigenic Variation: Application to Rapid Estimation of Influenza Mortality Burden
Source: PLoS Comput Biol. 2010 Aug 12;6(8):e1000882. doi: 10.1371/journal.pcbi.1000882 (PMC2920844; doi:10.1371/journal.pcbi.1000882)
Supplement: Table S3 — The Spearman and Pearson Correlation Coefficients between the excess all-cause mortalities and the integrated genetic distances relative to the previous 1–5 antigenic strains as background strains. The numbers in parenthesis are the P-values of the corresponding coefficients. The largest coefficient for each (sub)type is highlighted in bold. a: Not applicable due to limited number of antigenic strains. (0.03 MB DOC) [file pcbi.1000882.s007.doc]

| **Virus (sub)type** | **Variation** | **No. of background strains** | | | | |
| --- | --- | --- | --- | --- | --- | --- |
|  |  | **1** | **2** | **3** | **4** | **5** |
| A(H1N1) | Spearman | **0.52(0.23)** | 0.49(0.27) | 0.14(0.8) | -^a^ | - |
|  | Pearson | **0.61(0.15)** | 0.41(0.37) | 0.21(0.68) | - | - |
| A(H3N2) | Spearman | 0.51(0.06) | **0.64(0.02)** | 0.43(0.13) | 0.08(0.78) | 0.18(0.53) |
|  | Pearson | 0.42(0.13) | **0.5(0.07)** | 0.33(0.25) | 0.11(0.7) | 0.08(0.78) |
| B | Spearman | -0.02(0.95) | -0.05(0.91) | 0.38(0.36) | 0.54(0.24) | **0.77(0.10)** |
|  | Pearson | -0.08(0.84) | 0.09(0.82) | 0.56(0.16) | 0.66(0.11) | **0.75(0.09)** |
