# Supplementary material for: Correlation of Influenza Virus Excess Mortality with Antigenic Variation: Application to Rapid Estimation of Influenza Mortality Burden
Source: PLoS Comput Biol. 2010 Aug 12;6(8):e1000882. doi: 10.1371/journal.pcbi.1000882 (PMC2920844; doi:10.1371/journal.pcbi.1000882)
Supplement: Table S4 — The classical and robust regression analysis of the relationship between the antigenic distance and the excess mortality for human A(H1N1) using five different equations. The table lists the function, R-squared and P-value for each regression. (0.04 MB DOC) [file pcbi.1000882.s008.doc]

| **Fitting Model** | **Regression type** | **Function** | **R-squared** | **P-value** |
| --- | --- | --- | --- | --- |
| **Linear** | **Classical** | **y=31.31x-8.85** | **0.83** | **0.005** |
|  | **Robust** | **y=31.31x-8.15** | **0.51** | **2.51e-05** |
| Polynomial | Classical | y=5.02x^2^+10.48x+6.21 | 0.84 | 0.03 |
|  | Robust | y=-11.61x^2^+79.44x-36.25 | 0.54 | 0.02 |
| Logarithm | Classical | y=50.24ln(x)+28.05 | 0.76 | 0.01 |
|  | Robust | y=45.98ln(x)+31.93 | 0.57 | 7.49e-06 |
| Power | Classical | y=16.67x^1.49^ | 0.70 | 0.02 |
|  | Robust | y=30.56x^0.99^ | 0.48 | 0.001 |
| Exponential | Classical | y=6.70e^0.82x^ | 0.60 | 0.04 |
|  | Robust | y=14.24e^0.55x^ | 0.39 | 0.003 |
