# Supplementary material for: Correlation of Influenza Virus Excess Mortality with Antigenic Variation: Application to Rapid Estimation of Influenza Mortality Burden
Source: PLoS Comput Biol. 2010 Aug 12;6(8):e1000882. doi: 10.1371/journal.pcbi.1000882 (PMC2920844; doi:10.1371/journal.pcbi.1000882)
Supplement: Table S5 — The performance comparison between the EADpred method and one of the best site-based methods in predicting antigenic variants (see Methods). a: Based on the method proposed by Liao et al [11]. (0.03 MB DOC) [file pcbi.1000882.s009.doc]

| **Method** | **Dataset** | **Agreement** | **Sensitivity** | **Specificity** | **Coefficient** |
| --- | --- | --- | --- | --- | --- |
| Site-Based^a^ | Training | 0.81 | 0.82 | 0.79 | 0.82 |
|  | Testing | 0.84 | 0.81 | 0.85 | 0.75 |
| Epitope-Based (EADpred) | Training | 0.84 | 0.85 | 0.83 | 0.79 |
|  | Testing | 0.87 | 0.81 | 0.91 | 0.80 |
