# Supplementary material for: Correlation of Influenza Virus Excess Mortality with Antigenic Variation: Application to Rapid Estimation of Influenza Mortality Burden
Source: PLoS Comput Biol. 2010 Aug 12;6(8):e1000882. doi: 10.1371/journal.pcbi.1000882 (PMC2920844; doi:10.1371/journal.pcbi.1000882)
Supplement: Table S6 — The confidence interval of the Spearman and Pearson Correlation Coefficients between the excess all-cause mortalities and antigenic distances to previous individual antigenic strains. The numbers in parenthesis are the 95% confidence interval of corresponding coefficients. The numbers in red are the coefficients with P-value smaller than 0.05. a: The previous i-th antigenic strain is the i-th antigenic strain prior to an antigenic strain that is considered as a challenging strain. b: Not applicable due to the limited number of antigenic strains. (0.03 MB DOC) [file pcbi.1000882.s010.doc]

| Virus (sub)type | Variation | Previous antigenic strain^a^ | | | | |
| --- | --- | --- | --- | --- | --- | --- |
|  |  | 1^st^ | 2^nd^ | 3^rd^ | 4^th^ | 5^th^ |
| A(H1N1) | Spearman | 0.64(-0.25,0.94) | 0.64(-0.25,0.94) | -0.12(-0.86,0.78) | -^b^ | -^b^ |
|  | Pearson | **0.79(0.09,0.97)** | 0.65(-0.20,0.94) | -0.25(-0.88,0.70) | -^b^ | -^b^ |
| A(H3N2) | Spearman | **0.71(0.27,0.90)** | 0.21(-0.38,0.68) | -0.12(-0.62,0.45) | 0.13(-0.44,0.63) | 0.02(-0.51,0.56) |
|  | Pearson | **0.58(0.07,0.85)** | 0.31(-0.26,0.72) | -0.08(-0.59,0.47) | 0.19(-0.38,0.65) | 0.17(-0.40,0.64) |
| B | Spearman | 0.26(-0.46,0.77) | 0.57(-0.17,0.90) | **0.74(0.05,0.95)** | 0.07(-0.73,0.79) | -0.58(-0.95,0.46) |
|  | Pearson | 0.36(-0.35,0.81) | 0.53(-0.21,0.88) | **0.84(0.33,0.97)** | 0.14(-0.69,0.81) | -0.5(-0.93,0.52) |
