# Supplementary material for: Correlation of Influenza Virus Excess Mortality with Antigenic Variation: Application to Rapid Estimation of Influenza Mortality Burden
Source: PLoS Comput Biol. 2010 Aug 12;6(8):e1000882. doi: 10.1371/journal.pcbi.1000882 (PMC2920844; doi:10.1371/journal.pcbi.1000882)
Supplement: Table S7 — The confidence interval of the Spearman and Pearson Correlation Coefficients between the excess all-cause mortalities and the integrated antigenic distances relative to the previous 1–5 antigenic strains as background strains. The numbers in parenthesis are the 95% confidence interval of corresponding coefficients. The numbers in red are the coefficients with P-value smaller than 0.05. a: Not applicable due to limited number of antigenic strains. (0.03 MB DOC) [file pcbi.1000882.s011.doc]

| Virus (sub)type | Variation | No. of background strains | | | | |
| --- | --- | --- | --- | --- | --- | --- |
|  |  | 1 | 2 | 3 | 4 | 5 |
| A(H1N1) | Spearman | 0.64(-0.25,0.94) | 0.75(-0.04,0.96) | **0.94(0.52,0.99)** | -^a^ | -^a^ |
|  | Pearson | **0.79(0.09,0.97)** | **0.91(0.50,0.99)** | **0.85(0.12,0.98)** | -^a^ | -^a^ |
| A(H3N2) | Spearman | **0.71(0.27,0.90)** | 0.53(-0.02,0.83) | 0.57(0.04,0.85) | 0.28(-0.31,0.71) | 0.27(-0.32,0.71) |
|  | Pearson | **0.58(0.07,0.85)** | **0.57(0.06,0.85)** | 0.51(-0.03,0.82) | 0.39(-0.18,0.76) | 0.34(-0.23,0.74) |
| B | Spearman | 0.26(-0.46,0.77) | 0.55(-0.20,0.89) | **0.74(0.05,0.95)** | **0.79(0.06,0.97)** | 0.71(-0.27,0.97) |
|  | Pearson | 0.36(-0.35,0.81) | 0.48(-0.27,0.87) | **0.74(0.07,0.95)** | **0.78(0.07,0.97)** | 0.67(-0.31,0.96) |
