# Supplementary material for: Correlation of Influenza Virus Excess Mortality with Antigenic Variation: Application to Rapid Estimation of Influenza Mortality Burden
Source: PLoS Comput Biol. 2010 Aug 12;6(8):e1000882. doi: 10.1371/journal.pcbi.1000882 (PMC2920844; doi:10.1371/journal.pcbi.1000882)
Supplement: Table S8 — The classical and robust regression analysis of the relationship between the antigenic distance and the excess mortality for human A(H3N2) using five different equations. The table lists the function, R-squared and P-value for each regression. (0.04 MB DOC) [file pcbi.1000882.s012.doc]

| **Fitting model** | **Regression type** | **Function** | **R-squared** | **P-value** |
| --- | --- | --- | --- | --- |
| Linear | Classical | y=120.26x+47.37 | 0.33 | 0.03 |
|  | Robust | y=74.23x+97.04 | 0.26 | 0.02 |
| Polynomial | Classical | y=-40.67x^2^+267.89x-62.79 | 0.35 | 0.09 |
|  | Robust | y=-72.57x^2^ +337.86x-86.81 | 0.30 | 0.04 |
| **Logarithm** | **Classical** | **y=193.3ln(x)+170.8** | **0.35** | **0.03** |
|  | **Robust** | **y=126.42ln(x)+169.98** | **0.33** | **0.002** |
| Power | Classical | y=138.21x^0.96^ | 0.51 | 0.004 |
|  | Robust | y=223.63x^0.27^ | 0.04 | 0.29 |
| Exponential | Classical | y=79.05e^0.57x^ | 0.44 | 0.01 |
|  | Robust | y=196.37e^0.14x^ | 0.03 | 0.31 |
