# Supplementary material for: Correlation of Influenza Virus Excess Mortality with Antigenic Variation: Application to Rapid Estimation of Influenza Mortality Burden
Source: PLoS Comput Biol. 2010 Aug 12;6(8):e1000882. doi: 10.1371/journal.pcbi.1000882 (PMC2920844; doi:10.1371/journal.pcbi.1000882)
Supplement: Table S9 — The classical and robust regression analysis of the relationship between the antigenic distance and the excess mortality for human B virus using five different equations. The table lists the function, R-squared and P-value for each regression. (0.04 MB DOC) [file pcbi.1000882.s013.doc]

| **Fitting model** | **Regression type** | **Function** | **R-squared** | **P-value** |
| --- | --- | --- | --- | --- |
| Linear | Classical | y=34.86x-6.95 | 0.71 | 0.009 |
|  | Robust | y=34.86x-6.95 | 0.70 | 0.002 |
| Polynomial | Classical | y=-16.32x^2^+93.58x-51.82 | 0.78 | 0.02 |
|  | Robust | y=-16.32x^2^+93.58x-51.82 | 0.73 | 0.03 |
| **Logarithm** | **Classical** | **y=56.68ln(x)+27.64** | **0.77** | **0.004** |
|  | **Robust** | **y=56.68ln(x)+27.64** | **0.64** | **7.89e-05** |
| Power | Classical | y=18.94x^1.67^ | 0.85 | 0.001 |
|  | Robust | y=18.94x^1.67^ | 0.74 | 0.002 |
| Exponential | Classical | y=7.461e^0.98x^ | 0.71 | 0.009 |
|  | Robust | y=7.461e^0.98x^ | 0.53 | 0.008 |
