# Supplementary material for: Correlation of Influenza Virus Excess Mortality with Antigenic Variation: Application to Rapid Estimation of Influenza Mortality Burden
Source: PLoS Comput Biol. 2010 Aug 12;6(8):e1000882. doi: 10.1371/journal.pcbi.1000882 (PMC2920844; doi:10.1371/journal.pcbi.1000882)
Supplement: Table S10 — Antigenic distances between antigenic strains for human influenza A(H1N1), A(H3N2) and B, and antigenic distances between A(H1N1) viruses used for developing the EADpred method. (0.54 MB DOC) [file pcbi.1000882.s014.doc]

**Part I. Antigenic distances between antigenic strains of A(H1N1), A(H3N2) and B.**

**1. Antigenic distances between antigenic strains of A(H1N1) used in this study.**

| **Antigenic strain1** | **Antigenic strain2** | **Antigenic distance** |
| --- | --- | --- |
| A/England/333/1980 | A/Ussr/90/1977 | 1.039720771 |
| A/England/333/1980 | A/Brazil/11/1978 | 0.534799997 |
| A/Chile/1/1983 | A/Ussr/90/1977 | 2.426015132 |
| A/Chile/1/1983 | A/Brazil/11/1978 | 1.386294361 |
| A/Chile/1/1983 | A/England/333/1980 | 0.881373587 |
| A/Taiwan/1/1986 | A/Brazil/11/1978 | 3.119162313 |
| A/Taiwan/1/1986 | A/England/333/1980 | 3.50537720057182^a^ |
| A/Taiwan/1/1986 | A/Chile/1/1983 | 2.426015132 |
| A/Texas/36/1991 | A/England/333/1980 | 3.70958853320363^a^ |
| A/Texas/36/1991 | A/Chile/1/1983 | 3.119162313 |
| A/Texas/36/1991 | A/Taiwan/1/1986 | 0.752038699 |
| A/Bayern/7/1995 | A/Chile/1/1983 | 3.119162313 |
| A/Bayern/7/1995 | A/Taiwan/1/1986 | 0.534799997 |
| A/Bayern/7/1995 | A/Texas/36/1991 | 0.534799997 |
| A/New_caledonia/20/1999 | A/Taiwan/1/1986 | 2.772588722 |
| A/New_caledonia/20/1999 | A/Texas/36/1991 | 3.465735903 |
| A/New_caledonia/20/1999 | A/Bayern/7/1995 | 3.307388719 |
| A/Solomon_islands/3/2006 | A/Texas/36/1991 | 3.62859852322901^a^ |
| A/Solomon_islands/3/2006 | A/Bayern/7/1995 | 3.49559805708364^a^ |
| A/Solomon_islands/3/2006 | A/New_caledonia/20/1999 | 1.732867951 |
| A/Brisbane/59/2007 | A/Bayern/7/1995 | 3.65377027700037^a^ |
| A/Brisbane/59/2007 | A/New_caledonia/20/1999 | 2.426015132 |
| A/Brisbane/59/2007 | A/Solomon_islands/3/2006 | 0.693147181 |

**2. Antigenic distance between antigenic strains of A(H3N2) used in this study.**

| **Antigenic strain1** | **Antigenic strain2** | **Antigenic distance** |
| --- | --- | --- |
| A/Bangkok/1/1979 | A/Hong_kong/1/1968 | 3.465735903 |
| A/Bangkok/1/1979 | A/England/42/1972 | 3.664241451 |
| A/Bangkok/1/1979 | A/Port_chalmers/1/1973 | 3.321894867 |
| A/Bangkok/1/1979 | A/Victoria/3/1975 | 3.431067671 |
| A/Bangkok/1/1979 | A/Texas/1/1977 | 1.732867951 |
| A/Philippines/2/1982 | A/England/42/1972 | 3.97508858 |
| A/Philippines/2/1982 | A/Port_chalmers/1/1973 | 3.812309493 |
| A/Philippines/2/1982 | A/Victoria/3/1975 | 3.17805383 |
| A/Philippines/2/1982 | A/Texas/1/1977 | 1.732867951 |
| A/Philippines/2/1982 | A/Bangkok/1/1979 | 1.791759469 |
| A/Mississippi/1/1985 | A/Port_chalmers/1/1973 | 3.81231316127913^a^ |
| A/Mississippi/1/1985 | A/Victoria/3/1975 | 3.119162313 |
| A/Mississippi/1/1985 | A/Texas/1/1977 | 3.465735903 |
| A/Mississippi/1/1985 | A/Bangkok/1/1979 | 2.649158683 |
| A/Mississippi/1/1985 | A/Philippines/2/1982 | 0.693147181 |
| A/Shanghai/11/1987 | A/Victoria/3/1975 | 2.772588722 |
| A/Shanghai/11/1987 | A/Texas/1/1977 | 3.119162313 |
| A/Shanghai/11/1987 | A/Bangkok/1/1979 | 3.628514989 |
| A/Shanghai/11/1987 | A/Philippines/2/1982 | 2.935367809 |
| A/Shanghai/11/1987 | A/Mississippi/1/1985 | 2.035601227 |
| A/Beijing/353/1989 | A/Texas/1/1977 | 3.8663768935424^a^ |
| A/Beijing/353/1989 | A/Bangkok/1/1979 | 3.96609443721587^a^ |
| A/Beijing/353/1989 | A/Philippines/2/1982 | 3.79303674618851^a^ |
| A/Beijing/353/1989 | A/Mississippi/1/1985 | 3.80470501961953^a^ |
| A/Beijing/353/1989 | A/Shanghai/11/1987 | 2.020550024 |
| A/Beijing/32/1992 | A/Bangkok/1/1979 | 3.119162313 |
| A/Beijing/32/1992 | A/Philippines/2/1982 | 2.772588722 |
| A/Beijing/32/1992 | A/Mississippi/1/1985 | 3.119162313 |
| A/Beijing/32/1992 | A/Shanghai/11/1987 | 2.772588722 |
| A/Beijing/32/1992 | A/Beijing/353/1989 | 3.119162313 |
| A/Shangdong/9/1993 | A/Philippines/2/1982 | 2.96081468864994^a^ |
| A/Shangdong/9/1993 | A/Mississippi/1/1985 | 2.96081468864994^a^ |
| A/Shangdong/9/1993 | A/Shanghai/11/1987 | 2.77258872223978^a^ |
| A/Shangdong/9/1993 | A/Beijing/353/1989 | 3.465735903 |
| A/Shangdong/9/1993 | A/Beijing/32/1992 | 0.693147181 |
| A/Johannesburg/33/1994 | A/Mississippi/1/1985 | 3.119162313 |
| A/Johannesburg/33/1994 | A/Shanghai/11/1987 | 2.772588722 |
| A/Johannesburg/33/1994 | A/Beijing/353/1989 | 3.81231316127913^a^ |
| A/Johannesburg/33/1994 | A/Beijing/32/1992 | 2.079441542 |
| A/Johannesburg/33/1994 | A/Shangdong/9/1993 | 1.732867951 |
| A/Wuhan/359/1995 | A/Shanghai/11/1987 | 3.119162313 |
| A/Wuhan/359/1995 | A/Beijing/353/1989 | 3.8675819700725^a^ |
| A/Wuhan/359/1995 | A/Beijing/32/1992 | 3.119162313 |
| A/Wuhan/359/1995 | A/Shangdong/9/1993 | 3.11914388346919^a^ |
| A/Wuhan/359/1995 | A/Johannesburg/33/1994 | 2.772588722 |
| A/Sydney/5/1997 | A/Beijing/353/1989 | 4.15888308335967^a^ |
| A/Sydney/5/1997 | A/Beijing/32/1992 | 3.465735903 |
| A/Sydney/5/1997 | A/Shangdong/9/1993 | 3.46573590279973^a^ |
| A/Sydney/5/1997 | A/Johannesburg/33/1994 | 3.465735903 |
| A/Sydney/5/1997 | A/Wuhan/359/1995 | 2.614241538 |
| A/Panama/2007/1999 | A/Beijing/32/1992 | 3.48296168346479^a^ |
| A/Panama/2007/1999 | A/Shangdong/9/1993 | 3.48296168346479^a^ |
| A/Panama/2007/1999 | A/Johannesburg/33/1994 | 3.37562655021714^a^ |
| A/Panama/2007/1999 | A/Wuhan/359/1995 | 2.03559414189956^a^ |
| A/Panama/2007/1999 | A/Sydney/5/1997 | 1.039720771 |
| A/Fujian/411/2002 | A/Shangdong/9/1993 | 3.87120101090789^a^ |
| A/Fujian/411/2002 | A/Johannesburg/33/1994 | 3.87120101090789^a^ |
| A/Fujian/411/2002 | A/Wuhan/359/1995 | 3.20502027857776^a^ |
| A/Fujian/411/2002 | A/Sydney/5/1997 | 2.772588722 |
| A/Fujian/411/2002 | A/Panama/2007/1999 | 2.48490665 |
| A/California/7/2004 | A/Johannesburg/33/1994 | 4.01920426252982^a^ |
| A/California/7/2004 | A/Wuhan/359/1995 | 3.68026243400682^a^ |
| A/California/7/2004 | A/Sydney/5/1997 | 3.28719593676983^a^ |
| A/California/7/2004 | A/Panama/2007/1999 | 3.465735903 |
| A/California/7/2004 | A/Fujian/411/2002 | 0.995880456 |
| A/Wisconsin/67/2005 | A/Wuhan/359/1995 | 4.58665992322899^a^ |
| A/Wisconsin/67/2005 | A/Sydney/5/1997 | 4.0719114783064^a^ |
| A/Wisconsin/67/2005 | A/Panama/2007/1999 | 4.505456674 |
| A/Wisconsin/67/2005 | A/Fujian/411/2002 | 2.079441542 |
| A/Wisconsin/67/2005 | A/California/7/2004 | 1.039720771 |
| A/Brisbane/10/2007 | A/Sydney/5/1997 | 3.8516571001417^a^ |
| A/Brisbane/10/2007 | A/Panama/2007/1999 | 4.158883083 |
| A/Brisbane/10/2007 | A/Fujian/411/2002 | 2.426015132 |
| A/Brisbane/10/2007 | A/California/7/2004 | 0.881373587 |
| A/Brisbane/10/2007 | A/Wisconsin/67/2005 | 0.188226406 |

**3. Antigenic distance between antigenic strains of influenza B used in this study.**

| Antigenic strain1 | Antigenic strain2 | Antigenic distance |
| --- | --- | --- |
| B/Singapore/222/1979 | B/HongKong/5/1972 | 0.895879735 |
| B/USSR/100/1983 | B/HongKong/5/1972 | 1.66352829133097^a^ |
| B/USSR/100/1983 | B/Singapore/222/1979 | 1.039720771 |
| B/AnnArbor/1/1986 | B/HongKong/5/1972 | 1.66392609771817^a^ |
| B/AnnArbor/1/1986 | B/Singapore/222/1979 | 1.04027671165515^a^ |
| B/AnnArbor/1/1986 | B/USSR/100/1983 | 0.693147181 |
| B/Victoria/2/1987 | B/HongKong/5/1972 | 2.34650628005197^a^ |
| B/Victoria/2/1987 | B/Singapore/222/1979 | 2.079441542 |
| B/Victoria/2/1987 | B/USSR/100/1983 | 1.039720771 |
| B/Victoria/2/1987 | B/AnnArbor/1/1986 | 1.039720771 |
| B/Yamagata/16/1988 | B/HongKong/5/1972 | 2.6217658325052^a^ |
| B/Yamagata/16/1988 | B/Singapore/222/1979 | 2.426015132 |
| B/Yamagata/16/1988 | B/USSR/100/1983 | 2.772588722 |
| B/Yamagata/16/1988 | B/AnnArbor/1/1986 | 3.119162313 |
| B/Yamagata/16/1988 | B/Victoria/2/1987 | 2.772588722 |
| B/Panama/45/1990 | B/Singapore/222/1979 | 1.732867951 |
| B/Panama/45/1990 | B/USSR/100/1983 | 1.732867951 |
| B/Panama/45/1990 | B/AnnArbor/1/1986 | 1.73342389221509^a^ |
| B/Panama/45/1990 | B/Victoria/2/1987 | 2.138333059 |
| B/Panama/45/1990 | B/Yamagata/16/1988 | 1.574520768 |
| B/Beijing/184/1993 | B/USSR/100/1983 | 1.73342389221509^a^ |
| B/Beijing/184/1993 | B/AnnArbor/1/1986 | 1.73342389221509^a^ |
| B/Beijing/184/1993 | B/Victoria/2/1987 | 2.80578168959555^a^ |
| B/Beijing/184/1993 | B/Yamagata/16/1988 | 1.57484646766448^a^ |
| B/Beijing/184/1993 | B/Panama/45/1990 | 0.693147181 |
| B/Sichuan/379/1999 | B/AnnArbor/1/1986 | 2.08442908319087^a^ |
| B/Sichuan/379/1999 | B/Victoria/2/1987 | 3.91619429349696^a^ |
| B/Sichuan/379/1999 | B/Yamagata/16/1988 | 1.97546895129686^a^ |
| B/Sichuan/379/1999 | B/Panama/45/1990 | 0.657520002916794^a^ |
| B/Sichuan/379/1999 | B/Beijing/184/1993 | 1.039720771 |
| B/Shanghai/361/2002 | B/Victoria/2/1987 | 3.92448503081919^a^ |
| B/Shanghai/361/2002 | B/Yamagata/16/1988 | 1.98100146886658^a^ |
| B/Shanghai/361/2002 | B/Panama/45/1990 | 1.04027671165515^a^ |
| B/Shanghai/361/2002 | B/Beijing/184/1993 | 0.693147180559945^a^ |
| B/Shanghai/361/2002 | B/Sichuan/379/1999 | 0 |
| B/Florida/7/2004 | B/Yamagata/16/1988 | 1.98100146886658^a^ |
| B/Florida/7/2004 | B/Panama/45/1990 | 1.04027671165515^a^ |
| B/Florida/7/2004 | B/Beijing/184/1993 | 0.693147180559945^a^ |
| B/Florida/7/2004 | B/Sichuan/379/1999 | 0.34657359 |
| B/Florida/7/2004 | B/Shanghai/361/2002 | 0.34657359 |
| B/Florida/4/2006 | B/Panama/45/1990 | 1.04027671165515^a^ |
| B/Florida/4/2006 | B/Beijing/184/1993 | 0.693147180559945^a^ |
| B/Florida/4/2006 | B/Sichuan/379/1999 | 0.34657359 |
| B/Florida/4/2006 | B/Shanghai/361/2002 | 0.534799997 |
| B/Florida/4/2006 | B/Florida/7/2004 | 0 |

^a^ Using the distance between their antigenically similar strains to substitute it,because this distance is not available from HI data collected by us.

**Part II. Training and Testing Dataset^b^**

**1. Training Dataset**

| **Strain1** | **Strain2** | **Antigenic distance** |
| --- | --- | --- |
| A/Ussr/90/1977 | A/Arizona/14/1978 | 1.921094358 |
| A/Ussr/90/1977 | A/California/45/1978 | 2.772588722 |
| A/Ussr/90/1977 | A/Lackland/7/1978 | 0.405465108 |
| A/Ussr/90/1977 | A/Lackland/3/1978 | 1.732867951 |
| A/Ussr/90/1977 | A/India/6263/1980 | 2.426015132 |
| A/Ussr/90/1977 | A/Hong_Kong/2/1982 | 1.732867951 |
| A/Ussr/90/1977 | A/Chile/1/1983 | 2.426015132 |
| A/Ussr/90/1977 | A/Dunedin/27/1983 | 3.119162313 |
| A/Ussr/90/1977 | A/Victoria/7/1983 | 3.119162313 |
| A/Ussr/90/1977 | A/Singapore/6/1986 | 3.465735903 |
| A/Ussr/90/1977 | A/Taiwan/1/1986 | 3.119162313 |
| A/Ussr/90/1977 | A/Texas/36/1991 | 3.812309493 |
| A/Ussr/90/1977 | A/Bayern/7/1995 | 3.812309493 |
| A/Ussr/90/1977 | A/Beijing/262/1995 | 3.119162313 |
| A/Ussr/90/1977 | A/New_caledonia/20/1999 | 3.119162313 |
| A/Arizona/14/1978 | A/Brazil/11/1978 | 1.732867951 |
| A/Arizona/14/1978 | A/California/45/1978 | 2.426015132 |
| A/Arizona/14/1978 | A/Lackland/3/1978 | 1.921094358 |
| A/Arizona/14/1978 | A/Lackland/7/1978 | 2.079441542 |
| A/Brazil/11/1978 | A/California/45/1978 | 2.079441542 |
| A/Brazil/11/1978 | A/Lackland/3/1978 | 1.732867951 |
| A/Brazil/11/1978 | A/Lackland/7/1978 | 0.693147181 |
| A/Brazil/11/1978 | A/India/6263/1980 | 2.079441542 |
| A/Brazil/11/1978 | A/Hong_Kong/2/1982 | 1.039720771 |
| A/Brazil/11/1978 | A/Dunedin/27/1983 | 2.995732274 |
| A/Brazil/11/1978 | A/Victoria/7/1983 | 2.48490665 |
| A/Brazil/11/1978 | A/Singapore/6/1986 | 3.465735903 |
| A/Brazil/11/1978 | A/Taiwan/1/1986 | 3.119162313 |
| A/Brazil/11/1978 | A/Texas/36/1991 | 3.812309493 |
| A/Brazil/11/1978 | A/Bayern/7/1995 | 3.812309493 |
| A/Brazil/11/1978 | A/Beijing/262/1995 | 3.119162313 |
| A/Brazil/11/1978 | A/New_caledonia/20/1999 | 3.119162313 |
| A/California/45/1978 | A/Lackland/3/1978 | 1.386294361 |
| A/California/45/1978 | A/Lackland/7/1978 | 2.079441542 |
| A/Lackland/3/1978 | A/Lackland/7/1978 | 1.227947177 |
| A/Lackland/3/1978 | A/England/333/1980 | 2.426015132 |
| A/Lackland/3/1978 | A/India/6263/1980 | 1.732867951 |
| A/Lackland/3/1978 | A/Hong_Kong/2/1982 | 1.732867951 |
| A/Lackland/3/1978 | A/Chile/1/1983 | 1.732867951 |
| A/Lackland/3/1978 | A/Dunedin/27/1983 | 0.693147181 |
| A/Lackland/3/1978 | A/Victoria/7/1983 | 3.465735903 |
| A/England/333/1980 | A/India/6263/1980 | 1.386294361 |
| A/England/333/1980 | A/Hong_Kong/2/1982 | 0.34657359 |
| A/England/333/1980 | A/Dunedin/27/1983 | 2.614241538 |
| A/England/333/1980 | A/Victoria/7/1983 | 1.921094358 |
| A/India/6263/1980 | A/Hong_Kong/2/1982 | 0.693147181 |
| A/India/6263/1980 | A/Chile/1/1983 | 0.693147181 |
| A/India/6263/1980 | A/Dunedin/27/1983 | 1.039720771 |
| A/India/6263/1980 | A/Victoria/7/1983 | 2.772588722 |
| A/Hong_Kong/2/1982 | A/Chile/1/1983 | 0.34657359 |
| A/Hong_Kong/2/1982 | A/Dunedin/27/1983 | 1.386294361 |
| A/Hong_Kong/2/1982 | A/Victoria/7/1983 | 1.386294361 |
| A/Chile/1/1983 | A/Dunedin/27/1983 | 1.227947177 |
| A/Chile/1/1983 | A/Victoria/7/1983 | 1.791759469 |
| A/Chile/1/1983 | A/Singapore/6/1986 | 2.772588722 |
| A/Chile/1/1983 | A/Bayern/7/1995 | 3.119162313 |
| A/Chile/1/1983 | A/Beijing/262/1995 | 2.426015132 |
| A/Chile/1/1983 | A/New_caledonia/20/1999 | 2.426015132 |
| A/Dunedin/27/1983 | A/Victoria/7/1983 | 2.772588722 |
| A/Singapore/6/1986 | A/Taiwan/1/1986 | 0.693147181 |
| A/Singapore/6/1986 | A/Sichuan/4/1988 | 1.039720771 |
| A/Singapore/6/1986 | A/Texas/36/1991 | 0.34657359 |
| A/Singapore/6/1986 | A/Bayern/7/1995 | 0.693147181 |
| A/Singapore/6/1986 | A/Beijing/262/1995 | 3.119162313 |
| A/Singapore/6/1986 | A/New_caledonia/20/1999 | 3.119162313 |
| A/Taiwan/1/1986 | A/Sichuan/4/1988 | 0.693147181 |
| A/Taiwan/1/1986 | A/Beijing/262/1995 | 2.772588722 |
| A/Taiwan/1/1986 | A/Wuhan/371/1995 | 3.119162313 |
| A/Taiwan/1/1986 | A/New_caledonia/20/1999 | 2.772588722 |
| A/Texas/36/1991 | A/Beijing/262/1995 | 3.075321998 |
| A/Texas/36/1991 | A/Wuhan/371/1995 | 3.119162313 |
| A/Texas/36/1991 | A/Johannesburg/82/1996 | 1.039720771 |
| A/Bayern/7/1995 | A/Beijing/262/1995 | 2.960815129 |
| A/Bayern/7/1995 | A/Wuhan/371/1995 | 2.772588722 |
| A/Bayern/7/1995 | A/Johannesburg/82/1996 | 0.103893543 |
| A/Bayern/7/1995 | A/Singapore/9/1998 | 2.960815129 |
| A/Bayern/7/1995 | A/Hong_Kong/1252/2000 | 2.772588722 |
| A/Bayern/7/1995 | A/Madagascar/57794/2000 | 3.524627421 |
| A/Bayern/7/1995 | A/Egypt/96/2002 | 3.653962309 |
| A/Beijing/262/1995 | A/Wuhan/371/1995 | 0 |
| A/Beijing/262/1995 | A/Johannesburg/82/1996 | 3.119162313 |
| A/Beijing/262/1995 | A/Singapore/9/1998 | 0 |
| A/Beijing/262/1995 | A/New_caledonia/20/1999 | 1.039720771 |
| A/Beijing/262/1995 | A/Hong_Kong/1252/2000 | 2.138333059 |
| A/Beijing/262/1995 | A/Madagascar/57794/2000 | 1.227947177 |
| A/Beijing/262/1995 | A/Chile/9848/2002 | 1.039720771 |
| A/Beijing/262/1995 | A/Chile/8885/2002 | 1.227947177 |
| A/Beijing/262/1995 | A/Egypt/96/2002 | 1.732867951 |
| A/Beijing/262/1995 | A/Hawaii/10/2002 | 1.039720771 |
| A/Beijing/262/1995 | A/Neimenggu/52/2002 | 1.386294361 |
| A/Beijing/262/1995 | A/Hungary/2/2003 | 2.079441542 |
| A/Beijing/262/1995 | A/Peru/3135/2003 | 1.921094358 |
| A/Beijing/262/1995 | A/Hong_kong/2637/2004 | 1.574520768 |
| A/Beijing/262/1995 | A/Netherlands/128/2004 | 1.732867951 |
| A/Beijing/262/1995 | A/Egypt/39/2005 | 1.732867951 |
| A/Beijing/262/1995 | A/Jiangxi/160/2005 | 2.772588722 |
| A/Beijing/262/1995 | A/Kentucky/1/2005 | 1.039720771 |
| A/Beijing/262/1995 | A/Thessaloniki/24/2005 | 1.732867951 |
| A/Wuhan/371/1995 | A/Johannesburg/82/1996 | 2.809716569 |
| A/Wuhan/371/1995 | A/New_caledonia/20/1999 | 3.119162313 |
| A/Johannesburg/82/1996 | A/Singapore/9/1998 | 2.426015132 |
| A/Johannesburg/82/1996 | A/New_caledonia/20/1999 | 3.119162313 |
| A/Johannesburg/82/1996 | A/Hong_Kong/1252/2000 | 3.812309493 |
| A/Johannesburg/82/1996 | A/Chile/8885/2002 | 3.812309493 |
| A/Johannesburg/82/1996 | A/Hawaii/10/2002 | 4.0005359 |
| A/Johannesburg/82/1996 | A/Neimenggu/52/2002 | 4.34710949 |
| A/Singapore/9/1998 | A/New_caledonia/20/1999 | 0.693147181 |
| A/New_caledonia/20/1999 | A/Hong_Kong/1252/2000 | 1.227947177 |
| A/New_caledonia/20/1999 | A/Madagascar/57794/2000 | 0.188226406 |
| A/New_caledonia/20/1999 | A/Chile/9848/2002 | 0.34657359 |
| A/New_caledonia/20/1999 | A/Chile/8885/2002 | 0.34657359 |
| A/New_caledonia/20/1999 | A/Egypt/96/2002 | 0.34657359 |
| A/New_caledonia/20/1999 | A/Hawaii/10/2002 | 0.693147181 |
| A/New_caledonia/20/1999 | A/Neimenggu/52/2002 | 0.34657359 |
| A/New_caledonia/20/1999 | A/Hungary/2/2003 | 0.693147181 |
| A/New_caledonia/20/1999 | A/Peru/3135/2003 | 2.079441542 |
| A/New_caledonia/20/1999 | A/Brisbane/193/2004 | 0.693147181 |
| A/New_caledonia/20/1999 | A/Hong_kong/2637/2004 | 0 |
| A/New_caledonia/20/1999 | A/Netherlands/128/2004 | 0.693147181 |
| A/New_caledonia/20/1999 | A/New_caledonia/9/2004 | 0.188226406 |
| A/New_caledonia/20/1999 | A/Singapore/14/2004 | 0.534799997 |
| A/New_caledonia/20/1999 | A/Egypt/39/2005 | 0 |
| A/New_caledonia/20/1999 | A/Jiangxi/160/2005 | 2.079441542 |
| A/New_caledonia/20/1999 | A/Kentucky/1/2005 | 0.693147181 |
| A/New_caledonia/20/1999 | A/Shenzhen/141/2005 | 0.534799997 |
| A/New_caledonia/20/1999 | A/Thessaloniki/24/2005 | 0.34657359 |
| A/New_caledonia/20/1999 | A/Tokyo/6708/2005 | 2.079441542 |
| A/New_caledonia/20/1999 | A/Fukushima/141/2006 | 1.921094358 |
| A/New_caledonia/20/1999 | A/Hong_kong/2652/2006 | 2.079441542 |
| A/New_caledonia/20/1999 | A/Kentucky/2/2006 | -0.346573591 |
| A/New_caledonia/20/1999 | A/Malaysia/100/2006 | 0.693147181 |
| A/New_caledonia/20/1999 | A/Philippines/673/2006 | 2.138333059 |
| A/New_caledonia/20/1999 | A/Saint_petersburg/8/2006 | -0.346573591 |
| A/New_caledonia/20/1999 | A/Taiwan/42/2006 | 2.079441542 |
| A/New_caledonia/20/1999 | A/Victoria/500/2006 | 0.693147181 |
| A/New_caledonia/20/1999 | A/Virginia/1/2006 | 0 |
| A/New_caledonia/20/1999 | A/Cambodia/0371/2007 | 2.772588722 |
| A/New_caledonia/20/1999 | A/Egypt/10/2007 | 2.426015132 |
| A/New_caledonia/20/1999 | A/Netherlands/345/2007 | 2.079441542 |
| A/New_caledonia/20/1999 | A/South_dakota/6/2007 | 1.732867951 |
| A/New_caledonia/20/1999 | A/St_petersburg/10/2007 | 1.386294361 |
| A/New_caledonia/20/1999 | A/St_petersburg/12/2008 | 2.426015132 |
| A/New_caledonia/20/1999 | A/St_petersburg/5/2008 | 2.079441542 |

**2.** **Testing dataset**

| **Strain1** | **Strain2** | **Antigenic distance** |
| --- | --- | --- |
| A/Hong_Kong/1252/2000 | A/Madagascar/57794/2000 | 0 |
| A/Hong_Kong/1252/2000 | A/Egypt/96/2002 | 0.34657359 |
| A/Madagascar/57794/2000 | A/Egypt/96/2002 | 0.534799997 |
| A/Chile/9848/2002 | A/Chile/8885/2002 | 0 |
| A/Chile/9848/2002 | A/Hawaii/10/2002 | 0.693147181 |
| A/Chile/9848/2002 | A/Neimenggu/52/2002 | 0.34657359 |
| A/Chile/9848/2002 | A/Peru/3135/2003 | 2.426015132 |
| A/Chile/8885/2002 | A/Egypt/96/2002 | 0.693147181 |
| A/Chile/8885/2002 | A/Hawaii/10/2002 | 1.039720771 |
| A/Chile/8885/2002 | A/Neimenggu/52/2002 | 0.693147181 |
| A/Chile/8885/2002 | A/Hungary/2/2003 | 0.34657359 |
| A/Chile/8885/2002 | A/Peru/3135/2003 | 3.307388719 |
| A/Chile/8885/2002 | A/Hong_kong/2637/2004 | 0.34657359 |
| A/Chile/8885/2002 | A/Netherlands/128/2004 | 1.386294361 |
| A/Egypt/96/2002 | A/Hungary/2/2003 | 0.693147181 |
| A/Egypt/96/2002 | A/Hong_kong/2637/2004 | 0.34657359 |
| A/Egypt/96/2002 | A/Netherlands/128/2004 | 0.693147181 |
| A/Egypt/96/2002 | A/Egypt/39/2005 | 0.534799997 |
| A/Egypt/96/2002 | A/Thessaloniki/24/2005 | 0.693147181 |
| A/Egypt/96/2002 | A/Tokyo/6708/2005 | 2.772588722 |
| A/Egypt/96/2002 | A/Malaysia/100/2006 | 1.386294361 |
| A/Egypt/96/2002 | A/Taiwan/42/2006 | 2.772588722 |
| A/Egypt/96/2002 | A/Victoria/500/2006 | 1.386294361 |
| A/Hawaii/10/2002 | A/Neimenggu/52/2002 | 1.039720771 |
| A/Hawaii/10/2002 | A/Peru/3135/2003 | 3.119162313 |
| A/Neimenggu/52/2002 | A/Peru/3135/2003 | 2.614241538 |
| A/Neimenggu/52/2002 | A/Jiangxi/160/2005 | 2.079441542 |
| A/Neimenggu/52/2002 | A/Kentucky/1/2005 | 0.34657359 |
| A/Brisbane/193/2004 | A/New_caledonia/9/2004 | 0.188226406 |
| A/Brisbane/193/2004 | A/Singapore/14/2004 | -0.158347183 |
| A/Brisbane/193/2004 | A/Shenzhen/141/2005 | 0.058891518 |
| A/Brisbane/193/2004 | A/Fukushima/141/2006 | 3.465735903 |
| A/Brisbane/193/2004 | A/Hong_kong/2652/2006 | 2.426015132 |
| A/Brisbane/193/2004 | A/Malaysia/100/2006 | 0.188226406 |
| A/Brisbane/193/2004 | A/Philippines/673/2006 | 1.732867951 |
| A/Brisbane/193/2004 | A/Solomon_islands/3/2006 | 1.732867951 |
| A/Brisbane/193/2004 | A/Victoria/500/2006 | 0.405465108 |
| A/Hong_kong/2637/2004 | A/Netherlands/128/2004 | 0.693147181 |
| A/Hong_kong/2637/2004 | A/Egypt/39/2005 | -0.158347183 |
| A/Hong_kong/2637/2004 | A/Thessaloniki/24/2005 | 0.693147181 |
| A/Hong_kong/2637/2004 | A/Tokyo/6708/2005 | 1.386294361 |
| A/Hong_kong/2637/2004 | A/Malaysia/100/2006 | 0 |
| A/Hong_kong/2637/2004 | A/Taiwan/42/2006 | 1.732867951 |
| A/Hong_kong/2637/2004 | A/Victoria/500/2006 | 0.693147181 |
| A/Netherlands/128/2004 | A/Egypt/39/2005 | 0.534799997 |
| A/Netherlands/128/2004 | A/Thessaloniki/24/2005 | 0 |
| A/Netherlands/128/2004 | A/Tokyo/6708/2005 | 2.772588722 |
| A/Netherlands/128/2004 | A/Malaysia/100/2006 | 0.34657359 |
| A/Netherlands/128/2004 | A/Taiwan/42/2006 | 2.426015132 |
| A/Netherlands/128/2004 | A/Victoria/500/2006 | 0.34657359 |
| A/New_caledonia/9/2004 | A/Singapore/14/2004 | 0 |
| A/New_caledonia/9/2004 | A/Shenzhen/141/2005 | 0.188226406 |
| A/New_caledonia/9/2004 | A/Fukushima/141/2006 | 1.732867951 |
| A/New_caledonia/9/2004 | A/Hong_kong/2652/2006 | 1.732867951 |
| A/New_caledonia/9/2004 | A/Malaysia/100/2006 | -0.158347183 |
| A/New_caledonia/9/2004 | A/Philippines/673/2006 | 1.098612289 |
| A/New_caledonia/9/2004 | A/Solomon_islands/3/2006 | 1.039720771 |
| A/New_caledonia/9/2004 | A/Victoria/500/2006 | 0.188226406 |
| A/Singapore/14/2004 | A/Shenzhen/141/2005 | 0.058891518 |
| A/Singapore/14/2004 | A/Fukushima/141/2006 | 2.426015132 |
| A/Singapore/14/2004 | A/Hong_kong/2652/2006 | 1.732867951 |
| A/Singapore/14/2004 | A/Malaysia/100/2006 | -0.346573591 |
| A/Singapore/14/2004 | A/Philippines/673/2006 | 0.881373587 |
| A/Singapore/14/2004 | A/Solomon_islands/3/2006 | 0.881373587 |
| A/Singapore/14/2004 | A/Victoria/500/2006 | 0.058891518 |
| A/Egypt/39/2005 | A/Thessaloniki/24/2005 | 0.34657359 |
| A/Egypt/39/2005 | A/Tokyo/6708/2005 | 1.732867951 |
| A/Egypt/39/2005 | A/Fukushima/141/2006 | 1.227947177 |
| A/Egypt/39/2005 | A/Hong_kong/2652/2006 | 2.772588722 |
| A/Egypt/39/2005 | A/Malaysia/100/2006 | 0.693147181 |
| A/Egypt/39/2005 | A/Solomon_islands/3/2006 | 1.732867951 |
| A/Egypt/39/2005 | A/Taiwan/42/2006 | 2.426015132 |
| A/Egypt/39/2005 | A/Victoria/500/2006 | 0.693147181 |
| A/Egypt/39/2005 | A/St_petersburg/10/2007 | 1.039720771 |
| A/Jiangxi/160/2005 | A/Kentucky/1/2005 | 2.772588722 |
| A/Shenzhen/141/2005 | A/Fukushima/141/2006 | 3.812309493 |
| A/Shenzhen/141/2005 | A/Hong_kong/2652/2006 | 3.119162313 |
| A/Shenzhen/141/2005 | A/Malaysia/100/2006 | 0.058891518 |
| A/Shenzhen/141/2005 | A/Philippines/673/2006 | 1.732867951 |
| A/Shenzhen/141/2005 | A/Solomon_islands/3/2006 | 1.791759469 |
| A/Shenzhen/141/2005 | A/Victoria/500/2006 | 0.405465108 |
| A/Thessaloniki/24/2005 | A/Tokyo/6708/2005 | 2.426015132 |
| A/Thessaloniki/24/2005 | A/Fukushima/141/2006 | 2.614241538 |
| A/Thessaloniki/24/2005 | A/Hong_kong/2652/2006 | 2.772588722 |
| A/Thessaloniki/24/2005 | A/Malaysia/100/2006 | 0.34657359 |
| A/Thessaloniki/24/2005 | A/Solomon_islands/3/2006 | 2.426015132 |
| A/Thessaloniki/24/2005 | A/Taiwan/42/2006 | 2.079441542 |
| A/Thessaloniki/24/2005 | A/Victoria/500/2006 | 0.34657359 |
| A/Thessaloniki/24/2005 | A/Brisbane/59/2007 | 3.307388719 |
| A/Thessaloniki/24/2005 | A/Egypt/10/2007 | 2.772588722 |
| A/Thessaloniki/24/2005 | A/Netherlands/345/2007 | 2.426015132 |
| A/Thessaloniki/24/2005 | A/St_petersburg/10/2007 | 0.693147181 |
| A/Thessaloniki/24/2005 | A/St_petersburg/12/2008 | 2.772588722 |
| A/Thessaloniki/24/2005 | A/St_petersburg/5/2008 | 2.772588722 |
| A/Tokyo/6708/2005 | A/Malaysia/100/2006 | 2.426015132 |
| A/Tokyo/6708/2005 | A/Taiwan/42/2006 | 0.693147181 |
| A/Tokyo/6708/2005 | A/Victoria/500/2006 | 2.772588722 |
| A/Florida/03/2006 | A/Hong_kong/2652/2006 | 3.465735903 |
| A/Florida/03/2006 | A/Solomon_islands/3/2006 | 3.119162313 |
| A/Florida/03/2006 | A/Brisbane/59/2007 | 3.119162313 |
| A/Florida/03/2006 | A/South_dakota/6/2007 | 3.119162313 |
| A/Florida/03/2006 | A/Hong_kong/1870/2008 | 3.465735903 |
| A/Florida/03/2006 | A/Perth/200/2008 | 3.812309493 |
| A/Fukushima/141/2006 | A/Hong_kong/2652/2006 | 0.534799997 |
| A/Fukushima/141/2006 | A/Malaysia/100/2006 | 2.772588722 |
| A/Fukushima/141/2006 | A/Philippines/673/2006 | 1.039720771 |
| A/Fukushima/141/2006 | A/Solomon_islands/3/2006 | 0 |
| A/Fukushima/141/2006 | A/Victoria/500/2006 | 2.772588722 |
| A/Fukushima/141/2006 | A/Brisbane/59/2007 | 0.34657359 |
| A/Fukushima/141/2006 | A/Cambodia/0371/2007 | 0.693147181 |
| A/Fukushima/141/2006 | A/Egypt/10/2007 | 0.34657359 |
| A/Fukushima/141/2006 | A/Netherlands/345/2007 | 1.732867951 |
| A/Fukushima/141/2006 | A/South_dakota/6/2007 | 0 |
| A/Fukushima/141/2006 | A/St_petersburg/10/2007 | 2.772588722 |
| A/Fukushima/141/2006 | A/St_petersburg/5/2008 | 0.34657359 |
| A/Hong_kong/2652/2006 | A/Kentucky/2/2006 | 2.079441542 |
| A/Hong_kong/2652/2006 | A/Malaysia/100/2006 | 2.079441542 |
| A/Hong_kong/2652/2006 | A/Philippines/673/2006 | 1.039720771 |
| A/Hong_kong/2652/2006 | A/Saint_petersburg/8/2006 | 2.079441542 |
| A/Hong_kong/2652/2006 | A/Solomon_islands/3/2006 | 0.34657359 |
| A/Hong_kong/2652/2006 | A/Victoria/500/2006 | 2.426015132 |
| A/Hong_kong/2652/2006 | A/Virginia/1/2006 | 2.079441542 |
| A/Hong_kong/2652/2006 | A/Brisbane/59/2007 | 0.693147181 |
| A/Hong_kong/2652/2006 | A/Cambodia/0371/2007 | -0.346573591 |
| A/Hong_kong/2652/2006 | A/Egypt/10/2007 | 0.693147181 |
| A/Hong_kong/2652/2006 | A/Netherlands/345/2007 | 1.732867951 |
| A/Hong_kong/2652/2006 | A/South_dakota/6/2007 | 0.693147181 |
| A/Hong_kong/2652/2006 | A/St_petersburg/10/2007 | 2.079441542 |
| A/Hong_kong/2652/2006 | A/Hong_kong/1870/2008 | 0.34657359 |
| A/Hong_kong/2652/2006 | A/Perth/200/2008 | 0 |
| A/Hong_kong/2652/2006 | A/St_petersburg/12/2008 | 0.34657359 |
| A/Hong_kong/2652/2006 | A/St_petersburg/5/2008 | 0.34657359 |
| A/Kentucky/2/2006 | A/Saint_petersburg/8/2006 | 0 |
| A/Kentucky/2/2006 | A/Solomon_islands/3/2006 | 1.386294361 |
| A/Kentucky/2/2006 | A/Virginia/1/2006 | 0 |
| A/Malaysia/100/2006 | A/Philippines/673/2006 | 1.791759469 |
| A/Malaysia/100/2006 | A/Solomon_islands/3/2006 | 0.881373587 |
| A/Malaysia/100/2006 | A/Taiwan/42/2006 | 2.426015132 |
| A/Malaysia/100/2006 | A/Victoria/500/2006 | 0 |
| A/Philippines/673/2006 | A/Solomon_islands/3/2006 | 0 |
| A/Philippines/673/2006 | A/Victoria/500/2006 | 1.732867951 |
| A/Saint_petersburg/8/2006 | A/Solomon_islands/3/2006 | 1.732867951 |
| A/Saint_petersburg/8/2006 | A/Virginia/1/2006 | 0 |
| A/Solomon_islands/3/2006 | A/Victoria/500/2006 | 1.039720771 |
| A/Solomon_islands/3/2006 | A/Virginia/1/2006 | 0.693147181 |
| A/Solomon_islands/3/2006 | A/Brisbane/59/2007 | 0.693147181 |
| A/Solomon_islands/3/2006 | A/Egypt/10/2007 | 1.039720771 |
| A/Solomon_islands/3/2006 | A/Netherlands/345/2007 | 0.693147181 |
| A/Solomon_islands/3/2006 | A/South_dakota/6/2007 | 0.693147181 |
| A/Solomon_islands/3/2006 | A/St_petersburg/10/2007 | 2.772588722 |
| A/Solomon_islands/3/2006 | A/Hong_kong/1870/2008 | 1.039720771 |
| A/Solomon_islands/3/2006 | A/Perth/200/2008 | 0.34657359 |
| A/Solomon_islands/3/2006 | A/St_petersburg/12/2008 | 0.693147181 |
| A/Solomon_islands/3/2006 | A/St_petersburg/5/2008 | 0 |
| A/Taiwan/42/2006 | A/Victoria/500/2006 | 2.079441542 |
| A/Brisbane/59/2007 | A/Cambodia/0371/2007 | 1.039720771 |
| A/Brisbane/59/2007 | A/Egypt/10/2007 | 0.188226406 |
| A/Brisbane/59/2007 | A/Netherlands/345/2007 | 1.574520768 |
| A/Brisbane/59/2007 | A/South_dakota/6/2007 | 0.188226406 |
| A/Brisbane/59/2007 | A/Hong_kong/1870/2008 | 0.34657359 |
| A/Brisbane/59/2007 | A/Perth/200/2008 | 0.34657359 |
| A/Brisbane/59/2007 | A/St_petersburg/12/2008 | 0.34657359 |
| A/Brisbane/59/2007 | A/St_petersburg/5/2008 | 0 |
| A/Cambodia/0371/2007 | A/South_dakota/6/2007 | 1.039720771 |
| A/Egypt/10/2007 | A/Netherlands/345/2007 | 1.574520768 |
| A/Egypt/10/2007 | A/St_petersburg/12/2008 | 0 |
| A/Egypt/10/2007 | A/St_petersburg/5/2008 | 0.34657359 |
| A/Netherlands/345/2007 | A/St_petersburg/12/2008 | 1.039720771 |
| A/Netherlands/345/2007 | A/St_petersburg/5/2008 | 0.693147181 |
| A/South_dakota/6/2007 | A/Hong_kong/1870/2008 | 0.34657359 |
| A/South_dakota/6/2007 | A/Perth/200/2008 | 0.693147181 |
| A/Hong_kong/1870/2008 | A/Perth/200/2008 | 0.34657359 |
| A/St_petersburg/12/2008 | A/St_petersburg/5/2008 | 0 |

| **^b^All sequences collected from following references:** |
| --- |
| 1 Daniels, R.S., Douglas, A.R., Skehel, J.J., & Wiley, D.C., Antigenic and amino acid sequence analyses of influenza viruses of the H1N1 subtype isolated between 1982 and 1984. Bull World Health Organ 63 (2), 273-277 (1985). |
| 2 World Health Organization Collaborating Centre for Influenza - Australia. 2000. Information for the US consultation on formulation of influenza vaccines. |
| 3 WHO collaborating centre for reference and research on influenza-Lodon 2002. |
| 4 CDC. 2003. Information for the vaccines and related biological products advisory committee. |
| 5 WHO collaborating centre for reference and research on influenza-Lodon 2004. |
| 6 WHO collaborating centre for reference and research on influenza-Lodon 2006, September. |
| 7 WHO collaborating centre for reference and research on influenza-Lodon 2007, March. |
| 8 WHO collaborating centre for reference and research on influenza-Lodon 2008, March. |
| 9 Bao, Y. et al., The influenza virus resource at the National Center for Biotechnology Information. J Virol 82 (2), 596-601 (2008). |
| 10 Peter Bogner, et al.A global initiative on sharing avian flu data.  Nature,2006; 442. |

**Part III. The sources of the HI data for influenza virus A(H1N1), A(H3N2) and B.**

**1. The sources of the HI data for influenza virus A(H1N1).**

| 1 Ndifon, W., Dushoff, J., & Levin, S.A., On the use of hemagglutination-inhibition for influenza surveillance: surveillance data are predictive of influenza vaccine effectiveness. Vaccine 27 (18), 2447-2452 (2009). |
| --- |
| 2 Daniels, R.S., Douglas, A.R., Skehel, J.J., & Wiley, D.C., Antigenic and amino acid sequence analyses of influenza viruses of the H1N1 subtype isolated between 1982 and 1984. Bull World Health Organ 63 (2), 273-277 (1985). |
| 3 WHO collaborating centre for reference and research on influenza-Lodon 2002. |
| 4 Nakajima, S., Cox, N.J., & Kendal, A.P., Antigenic and genomic analyses of influenza A(H1N1) viruses from different regions of the world, February 1978 to March 1980. Infect Immun 32 (1), 287-294 (1981). |
| 5 Cox, N.J., Bai, Z.S., & Kendal, A.P., Laboratory-based surveillance of influenza A(H1N1) and A(H3N2) viruses in 1980-81: antigenic and genomic analyses. Bull World Health Organ 61 (1), 143-152 (1983). |
| 6 Kendal, A.P. et al., Laboratory-based surveillance of influenza virus in the United States during the winter of 1977-1978. I. Periods of prevalence of H1N1 and H3N2 influenza A strains, their relative rates of isolation in different age groups, and detection of antigenic variants. Am J Epidemiol 110 (4), 449-461 (1979). |
| 7 CDC.2009. Information for the vaccines and related biological products advisory committee. |
| 8 CDC.2004. Information for the vaccines and related biological products advisory committee. |
| 9 WHO collaborating centre for reference and research on influenza-Lodon 2003. |
| 10 WHO collaborating centre for reference and research on influenza-Lodon 2004. |
| 11 WHO collaborating centre for reference and research on influenza-Lodon 2005,February. |
| 12 WHO collaborating centre for reference and research on influenza-Lodon 2005, September. |
| 13 WHO collaborating centre for reference and research on influenza-Lodon 2006, March. |
| 14 WHO collaborating centre for reference and research on influenza-Lodon 2006, September. |
| 15 WHO collaborating centre for reference and research on influenza-Lodon 2007, March. |
| 16 WHO collaborating centre for reference and research on influenza-Lodon 2007, September. |
| 17 WHO collaborating centre for reference and research on influenza-Lodon 2008, March. |
| 18 WHO collaborating centre for reference and research on influenza-Lodon 2008, September. |
| 19 WHO collaborating centre for reference and research on influenza-Lodon 2009, February. |
| 20 WHO collaborating centre for reference and research on influenza- Australia,2006. |

**2. The sources of the HI data for influenza virus A(H3N2).**

| 1 Ndifon, W., Dushoff, J., & Levin, S.A., On the use of hemagglutination-inhibition for influenza surveillance: surveillance data are predictive of influenza vaccine effectiveness. Vaccine 27 (18), 2447-2452 (2009). |
| --- |
| 2 Cox, N.J., Bai, Z.S., & Kendal, A.P., Laboratory-based surveillance of influenza A(H1N1) and A(H3N2) viruses in 1980-81: antigenic and genomic analyses. Bull World Health Organ 61 (1), 143-152 (1983). |
| 3 Kendal, A.P. et al., Laboratory-based surveillance of influenza virus in the United States during the winter of 1977-1978. I. Periods of prevalence of H1N1 and H3N2 influenza A strains, their relative rates of isolation in different age groups, and detection of antigenic variants. Am J Epidemiol 110 (4), 449-461 (1979). |
| 4 WHO collaborating centre for reference and research on influenza-Lodon 2002. |
| 5 WHO collaborating centre for reference and research on influenza-Lodon 2003. |
| 6 WHO collaborating centre for reference and research on influenza-Lodon 2004. |
| 7 WHO collaborating centre for reference and research on influenza-Lodon 2005,February. |
| 8 WHO collaborating centre for reference and research on influenza-Lodon 2005, September. |
| 9 WHO collaborating centre for reference and research on influenza-Lodon 2006, March. |
| 10 WHO collaborating centre for reference and research on influenza-Lodon 2006, September. |
| 11 WHO collaborating centre for reference and research on influenza-Lodon 2007, March. |
| 12 WHO collaborating centre for reference and research on influenza-Lodon 2007, September. |
| 13 WHO collaborating centre for reference and research on influenza-Lodon 2008, March. |
| 14 WHO collaborating centre for reference and research on influenza-Lodon 2008, September. |
| 15 WHO collaborating centre for reference and research on influenza-Lodon 2009, February. |
| 16 WHO collaborating centre for reference and research on influenza- Australia,2006. |

**3. The sources of the HI data for influenza virus B.**

| 1 Ndifon, W., Dushoff, J., & Levin, S.A., On the use of hemagglutination-inhibition for influenza surveillance: surveillance data are predictive of influenza vaccine effectiveness. Vaccine 27 (18), 2447-2452 (2009). |
| --- |
| 2 WHO collaborating centre for reference and research on influenza-Lodon 2002. |
| 3 WHO collaborating centre for reference and research on influenza-Lodon 2003. |
| 4 WHO collaborating centre for reference and research on influenza-Lodon 2004. |
| 5 WHO collaborating centre for reference and research on influenza-Lodon 2005,February. |
| 6 WHO collaborating centre for reference and research on influenza-Lodon 2005, September. |
| 7 WHO collaborating centre for reference and research on influenza-Lodon 2006, March. |
| 8 WHO collaborating centre for reference and research on influenza-Lodon 2006, September. |
| 9 WHO collaborating centre for reference and research on influenza-Lodon 2007, March. |
| 10 WHO collaborating centre for reference and research on influenza-Lodon 2007, September. |
| 11 WHO collaborating centre for reference and research on influenza-Lodon 2008, March. |
| 12 WHO collaborating centre for reference and research on influenza-Lodon 2008, September. |
| 13 WHO collaborating centre for reference and research on influenza-Lodon 2009, February. |
| 14 WHO collaborating centre for reference and research on influenza- Australia,2006. |
