# Supplementary material for: Correlation of Influenza Virus Excess Mortality with Antigenic Variation: Application to Rapid Estimation of Influenza Mortality Burden
Source: PLoS Comput Biol. 2010 Aug 12;6(8):e1000882. doi: 10.1371/journal.pcbi.1000882 (PMC2920844; doi:10.1371/journal.pcbi.1000882)
Supplement: Table S11 — Six predicted epitopes of the A(H1N1) HA protein. a: The epitopes are extended from the known epitopes based on references 12–14. b: Two predicted novel antigenic eptiopes supported by references 17 and 18. (0.03 MB DOC) [file pcbi.1000882.s015.doc]

| **Epitope** | **Number** | **Sites** | **Reference** |
| --- | --- | --- | --- |
| Sa^a^ | 19 | 123 124 125 126 128 129 156 157 158 159 160 161 162 163 164 165 166 167 168 | 12-14 |
| Sb^a^ | 19 | 187 188 189 190 191 192 193 194 195 196 197 198 199 200 201 210 211 212 213 | 12-14 |
| Ca^a^ | 32 | 139 140 141 142 143 144 145 146 149 169 170 171 172 173 206 207 208 217 218 219 222 223 224 225 226 227 238 239 240 241 242 243 | 12-14 |
| Cb^a^ | 24 | 58 72 73 74 75 76 77 78 79 86 87 88 89 90 91 93 119 256 261 262 263 264 265 | 12-14 |
| Pa^b^ | 19 | 47 49 50 51 52 53 55 272 273 274 275 276 277 279 280 281 286 298 305 | 17 |
| Pb^b^ | 16 | 17 18 19 32 33 34 35 39 40 41 42 289 290 291 292 293 | 18 |
