# Supplementary material for: Correlation of Influenza Virus Excess Mortality with Antigenic Variation: Application to Rapid Estimation of Influenza Mortality Burden
Source: PLoS Comput Biol. 2010 Aug 12;6(8):e1000882. doi: 10.1371/journal.pcbi.1000882 (PMC2920844; doi:10.1371/journal.pcbi.1000882)
Supplement: Table S12 — Values of five selected physiochemical properties of the 20 amino acids. a: The hydrophobic values came from the BLAS910101 entry in AAindex database [21]. (0.05 MB DOC) [file pcbi.1000882.s016.doc]

| **Residue** | **hydrogen-**  **bond Donor** | **hydrogen-**  **bond Acceptor** | **Positive charge** | **Negative charge** | **Hydrophobicity^a^** |
| --- | --- | --- | --- | --- | --- |
| **A** | 0 | 0 | 0 | 0 | 0.616 |
| **R** | 1 | 0 | 1 | 0 | 0 |
| **N** | 1 | 1 | 0 | 0 | 0.236 |
| **D** | 1 | 1 | 0 | 1 | 0.028 |
| **C** | 0 | 0 | 0 | 0 | 0.68 |
| **Q** | 1 | 1 | 0 | 0 | 0.251 |
| **E** | 0 | 1 | 0 | 1 | 0.043 |
| **H** | 0 | 1 | 1 | 0 | 0.165 |
| **I** | 0 | 0 | 0 | 0 | 0.943 |
| **L** | 0 | 0 | 0 | 0 | 0.943 |
| **K** | 1 | 0 | 1 | 0 | 0.283 |
| **M** | 0 | 0 | 0 | 0 | 0.738 |
| **F** | 0 | 0 | 0 | 0 | 1 |
| **P** | 0 | 0 | 0 | 0 | 0.711 |
| **S** | 1 | 1 | 0 | 0 | 0.359 |
| **T** | 1 | 1 | 0 | 0 | 0.45 |
| **W** | 1 | 0 | 0 | 0 | 0.878 |
| **Y** | 1 | 1 | 0 | 0 | 0.88 |
| **V** | 0 | 0 | 0 | 0 | 0.825 |
| **G** | 0 | 0 | 0 | 0 | 0.501 |
