# Supplementary material for: Correlation of Influenza Virus Excess Mortality with Antigenic Variation: Application to Rapid Estimation of Influenza Mortality Burden
Source: PLoS Comput Biol. 2010 Aug 12;6(8):e1000882. doi: 10.1371/journal.pcbi.1000882 (PMC2920844; doi:10.1371/journal.pcbi.1000882)
Supplement: Text S1 — Supporting methods, legends for supporting tables and figures. (0.10 MB DOC) [file pcbi.1000882.s017.doc]

**Supporting Information**

**Correlation of Influenza Virus Excess Mortality with Antigenic Variation: Application to Rapid Estimation of Influenza Mortality Burden**

Aiping Wu, Yousong Peng, Xiangjun Du, Yuelong Shu, Taijiao Jiang

**SI Text**

**Hemagglutination inhibition (HI) data** used to calculate antigenic distances between viral strains were collected from the Reference *1* and other articles or documents published by the collaborating centers of the World Heath Organization (WHO)’s global influenza surveillance network (Table S10)**.** To calculate the antigenic distance between two strains *i* and *j, d_ij_,* we followed the Archetti-Horsfall method with adaptation [1,2]:

(1)

where H^ij^ refers to the HI titer of *i* relative to antisera raised against *j*. If the antigenic distance of a pair of viruses was measured in several independent HI assays, the median value was used of all antigenic distances calculated from these independent assays.

In developing the Epitope-based Antigenic Distance prediction (EADpred) model, only the 330 antigenic distances between strains of human A(H1N1) virus with HA1 peptide sequences (1-331 resides) available were considered. After excluding 15 antigenic distances between antigenic strains (see the definition of antigenic strain as follows), the remaining 315 antigenic distances were separated into a training data set and a testing data set. The training dataset consists of 143 antigenic distances between A(H1N1) strains, in which at least one strain in each pair was isolated between 1977 and 1999. The testing dataset consists of 172 antigenic distances between A(H1N1) strains that were both isolated between 2000 and 2008 (Table S10).

**HA sequences** of 2000 human A(H1N1) viruses isolated in USA from the year 1918 through 2008 were collected from the Influenza Virus Resource at the National Center for Biotechnology Information (NCBI) [3]. The strain A/Brisbane/59/2007 (Genbank sequence accession number: CY030230) was used as the representative strain of the seasonal A(H1N1) virus of 2008-2009 season. All these sequences were aligned using the software Clustal W 2.0.9 [4], and were renumbered according to the numbering of the HA1 peptide sequence of A/putertorico/8/34(H1N1) virus with known HA structure (PDB ID: 1RVZ).

**Surveillance data** for influenza viruses in the US were extracted from the reports of Centers for Disease Control and Prevention of the United States (US CDC) and related research articles (Table S1). These data that were arranged by flu seasons include the number of total respiratory specimens tested for influenza and positive isolates of three human influenza viruses, A(H1N1), A(H3N2) and B from season 1977-1978 through 2008-2009 (Table S1). A flu season is defined as the period from October 1 through September 30 of the following year. The specimens for season 1977-1978 to season 1998-1999 were from ref 33 cited in Table S1, and the specimens in the remaining seasons were collected from the reports of influenza surveillance by US CDC (see Table S1). The specimens for a flu season were sum of the weekly data.

**Mortality data** from 1977 to 2006 were extracted from the US national multiple-cause-of-death dataset provided by the National Center for Health Statistics (NCHS) [5]. The national multiple-cause-of-death data for year 2007 through 2009 is not available from NCHS, and are obtained based on the death data collected from US CDC 122 Cities Mortality Reporting System [6].

**The annual US human population** from 1977 to 2009 were obtained from the US Bureau of the Census [7].

**Identification of antigenic strains for each (sub)type of human influenza virus.** In this study, all analyses were performed for strains within same (sub)type since there was little cross immunity observed between (sub)types [8]. A strain is defined to be dominant in a season when the number of its antigenically similar isolates exceeds half of the isolates of the same (sub)type antigenically characterized by CDC during the season. To analyze the relationships between antigenic variation and excess all-cause mortality, we defined as “antigenic strains” the vaccine strains and reference strains that were used by the US CDC to characterize the antigenic properties of circulating strains in influenza surveillance. Only the strains that were dominant in at least one flu season were considered in our study (see Table S1 for their circulation time). We considered these vaccine strains and reference strains as antigenic strains in our study because of the following three reasons: First, they are good representatives of strains of significant impact due to their considerable antigenic changes and ability to infect the human population. Second, their antigenic distances were well determined. Third, their periods of dominance and circulation were well recorded so that their excess all-cause mortalities could easily be inferred.

For antigenic strains of human influenza A(H1N1) virus from 1977 through 2009, the strain A/USSR/90/1977 that appreared in season 1977-1978 was considered as the first antigenic strain since the Russian Flu. The most recent antigenic strain that has completed its circulation is A/Solomon_islands/3/2006 for the flu seasons in 2006-2007 and 2007-2008. The recent antigenic strain A/Brisbane/59/2007 started to circulate in 2007-2008, and is still in circulation. In the April of 2009, another A(H1N1) strain of novel antigenicity emerged and has spread to almost the whole world. For the influenza A(H3N2) virus circulated from 1977 through 2009, we considered A/Bangkok/1/1979 as the first antigenic strain that circulated from 1979 through 1983. The most recent antigenic strain that has completed its circulation is A/Wisconsin/67/2005, which is thus considered as the last antigenic strain in our analysis. For influenza B virus, B/Singapore/222/1979 is considered as the first antigenic strain, and B/Florida/7/2004 is considered as the last antigenic strain. Although two antigenically distinct lineages of B/Yamagata/16/1988 and B/Victoria/2/1987 have been co-circulating in the Asia since 1990, the viruses of the latter lineage, Victoria lineage, were not detected in the US until 2001 [9]. For simplicity, we didn’t consider the Victoria lineage that has been co-circulating with the Yamagata lineage since 2001 in the US. All the antigenic strains identified for the human influenza viruses, A(H1N1), A(H3N2) and B, are shown in Table S1.

**Calculation of integrated antigenic/genetic distance.** Integrated antigenic (or genetic) distance (D) is introduced to represent the antigenic (or genetic) variation of an antigenic strain as challenging strain relative to the previous antigenic strains, which is defined as a linear function of the antigenic (or genetic) distances between the challenging strain and its previous antigenic strains:

$D=\sum_{i} Wi*di$，i=1,2,..n, (2)

where d_i_ refers to the antigenic distance (or the number of amino acid differences on HA1 peptide) between the challenging antigenic strain and the *i*-th antigenic strain before the challenging antigenic strain. W_i_, a weight for d_i_, is calculated by $Wi=Ei/\sum_{i} Ei$, in which E_i_ refers to the number of epidemic seasons when the previous *i*-th antigenic strain was in circulation. An antigenic strain is defined to be epidemic in a season when the number of its antigenically similar isolates exceeds 10% of all isolates antigenically characterized by CDC in the season (Table S1).

**Epitope-based Antigenic Distance Prediction (EADpred)**. Although numerous methods have been developed to qualitatively identify antigenic variants [10,11] from HA sequences, the method is not available for predicting the quantitative changes of antigenicity, i.e., antigenic distances, between A(H1N1) viruses from their HA sequences. Here we proposed a novel method to predict antigenic distances between A(H1N1) viruses with high accuracy. Unlike numerous previous methods that considered specific amino acid positions of HA as variables for predicting antigenic variants [11], our method considers the antigenic epitopes on HA as basic structural units that mediate HA-antibody interactions. Another advantage of our method over the others is that our method considers the physicochemical rules underlying HA-antibody interactions rather than the specific amino acid changes. The development of the novel method consists of four steps described as follows:

*Step 1*. Identification of the antigenic epitopes as structural base underlying the HA-antibody interaction.

To define a comprehensive set of antigenic epitopes of the HA of A(H1N1) virus that may participate in the direct interactions with host neutralizing antibodies, we integrated the information from the following three sources:

1. Known antigenic sites and epitopes: Four epitopes of the HA of A(H1N1) virus were identified previously, including strain-specific epitopes Sa, Sb and cross-reactive epitopes, Ca and Cb [12-14].
2. Structure-based antigenic site predictions: A fragment consisting of at least three continuous amino acid residues is predicted to be a potential antigenic determinant (part of epitope) if each residue of the fragment has an Accessible Surface Area (ASA) ≥20Å^2^ [15]. The ASA for each residue was calculated using the naccess program [16] based on the X-ray structure of the HA of A/putertorico/8/34(H1N1) virus (PDB ID: 1RVZ). In total, 20 such fragments were predicted as potential antigenic determinants, and 11 of them are already included in the four known epitopes.
3. Hotspot residues subject to mutations: A fragment consisting of at least three continuous surface sites is predicted to be a potential antigenic determinant if at least three different types of amino acids occurred in each amino acid position of the fragment during evolution. A surface amino acid residue is a residue with an ASA≥1 Å^2^. The amino acids in each position of the HA of A(H1N1) virus were identified in a multiple-sequence alignment of 875 human A(H1N1) HA full-length sequences downloaded from the the Influenza Virus Resource at NCBI as of 16 May 2009. In total, 18 such fragments were predicted as potential antigenic determinants. Among them, 10 are already included in the four known epitopes and 5 are included in the 9 new fragments from the above structure-based prediction.

All these known and predicted epitopes were mapped onto the X-ray structure of the HA of A/putertorico/8/34(H1N1) virus (PDB ID: 1RVZ) (Figure 3A). The predicted antigenic determinants that overlapped with the four known epitopes were merged into the known epitopes. The remaining predicted fragments form two clusters on the structure, which were identified as two potential antigenic epitopes, Pa and Pb. Therefore, we have derived six antigenic epitopes in the HA of A(H1N1) virus, including four expanded known antigenic epitopes (Sa, Sb, Ca and Cb) and two novel antigenic epitopes (Pa and Pb) (Figure 3A). Interestingly, the predicted epitope Pa in the HA of A(H1N1) virus is similar to the epitope C in the HA of A(H3N2) virus [17], and the predicted epitope Pb is located at the interface of the complex of A(H1N1) HA with antibody based on the recently determined complex structure (PDB ID: 3gbn) [18]. These observations validate the reliability of our methods in prediction of the potential antigenic sites. The composition of the six antigenic epitopes and the supporting sources are summarized in Table S11.

*Step 2.* Transformation of amino acid changes in an antigenic epitope into the changes in physicochemical properties that underlie HA-antibody interaction.

To capture the physiochemical interactions between HA-antibodies that usually involve hydrogen-bond, salt-bridge and hydrophobic interactions, we used the five variables, hydrogen-bond donor (N_donor_), hydrogen-bond acceptor (N_acceptor_), positive charge (N_pc_), negative charge (N_nc_) and hydrophobicity (f_hydro_) to characterize the physiochemical properties of an amino acid. Therefore, the amino acid changes in an antigenic epitope were transformed into a linear combination of changes in the above five variables:

$f(E)=N_{donor}+a_{1}*N{}_{acceptor}+a_{2}*N_{pc}+a_{3}*N_{nc}+a_{4}*f_{hydro}$ (3)

where f(E) quantifies the changes in the physiochemical properties in the antigenic epitope. N_donor_ and N_acceptor_ represent the number of the changed hydrogen-bond donors and acceptors. N_pc_ and N_nc_ represent the number of the positive changes and negative charges. $f_{hydro}=\sum_{i} N_{i,hydro}$ represents the changed hydrophobicity of the epitope that consists of several cavities with at least two amino acids, which is a sum of hydrophobicities of these cavities.

To calculate the N_donor_, N_acceptor_, N_pc_, N_nc_ and f_hydro_, each of the 20 amino acids was assigned to a vector of values based on whether it is a hydrogen-bond donor or not (1, 0), whether it is a hydrogen-bond acceptor or not (1, 0), whether it is positively charged or not (1, 0), whether it is negatively charged or not (1, 0) and its hydrophobicity (0-1) (Table S12).

*Step 3.* Integration of the contributions of the six derived antigenic epitopes to predict the antigenic distance.

To predict the antigenic distances between two viral strains (d), we considered a linear combination of the changes in physicochemical properties in all the six derived antigenic epitopes:

$f(E_{1})+b_{1}*f(E_{2})+b_{2}*f(E_{3})+b_{3}*f(E_{4})+b_{4}*f(E_{5})+b_{5}*f(E{}_{6})+b_{6}=d$ (4)

where f(E_i_) denotes the function of the *i*-th epitope.

Then the equation 3 and 4 were combined into one equation, which is re-represented as follows:

$\begin{matrix} N_{donor}^{1}+\omega_{1}*N_{acceptor}^{1}+\omega_{2}*N_{pc}^{1}+\omega_{3}*N_{nc}^{1}+\omega_{4}*N_{1,hydro}^{1}+\omega_{5}*N_{2,hydro}^{1}+... \\ +\omega_{n}*N_{donor}^{i}+\omega_{n+1}*N_{acceptor}^{i}+\omega_{n+2}*N_{pc}^{i}+\omega_{n+3}*N_{nc}^{i}+\omega_{n+4}*N_{1,hydro}^{i}+\omega_{n+5}*N_{2,hydro}^{i}+... \\ +\omega_{42}*N_{donor}^{6}+\omega_{43}*N_{acceptor}^{6}+\omega_{44}*N_{pc}^{6}+\omega_{45}*N_{nc}^{6}+\omega_{46}*N_{1,hydro}^{6}+\omega_{47}*N_{2,hydro}^{6}+\omega_{48}=d \end{matrix}$ (5)

where $\omega_{n}$ are relative weights of the different terms. N^i^_donor_, N^i^_acceptor_, N^i^_pc_ and N^i^_nc_ are the four variables describing the effects of the hydrogen bonding and salt bridge of the *i*-th epitope that was described in equation 4. $N_{j,hydro}^{i}$ is the *j*-th cavity of the *i*-th epitope.

*Step 4*. Model parameterization and assessment.

The relative weights of the equation 5 were parameterized on the training dataset described above using a stepwise multiple regression. The individual variables in equation 5 and the antigenic distances were deemed independent variables and dependent variable. After regression, a modified item $f_{\mathrm{mod}ify}=\omega*(N_{f}\text{-N}_{\text{ave}})$ was added to adjust the predicted value slightly. Where $N_{f}$ is the number of features with non-zero weight, $N_{ave}$ is the average of $N_{f}$s over the training set, $\omega$ is the weight.

The prediction performance of the model parameterized on the training was further assessed using a testing dataset described above. This test is a blind test, because the testing data cover viruses isolated from 2000 through 2008 that were not included in the training set. The testing results were shown in Figure 3D and Table S5.

**Comparison of EADpred to One of the Best Existing Site-based Approaches.** Many methods have been developed for predicting antigenic variants qualitatively or semi-quantitatively from HA sequences [10,11,19,20]. These methods were dependent on the amount of genetic changes, particularly amino acid changes at specific positions on HA, for which we called site-based methods. To compare our model to one of the most recent site-dependent methods proposed by Liao et al [11], following the algorithm that Liao et al used for predicting antigenic variants of A(H3N2) virus, we implemented the algorithm to predict the antigenic variants of A(H1N1) virus (called Liao-like method). For comparison, a virus pair is regarded as an antigenic variant pair if the observed or predicted antigenic distance between the pair of viruses is ≥ln(4), otherwise, it is regarded as an antigenic similarity pair [11]. Both methods were trained on the same training data, and tested on the same testing data. Then, agreement rate, sensitivity and specificity were calculated for comparisons of EADpred method and the traditional site-based method, Liao-like method [11]. The agreement rate is defined as the ratio of all truly predicted pairs to the number of all virus pairs. The sensitivity and specificity were defined as the ratio of predicted antigenic variant pairs to true antigenic variant pairs and the ratio of predicted antigenic similarity pairs to true antigenic similarity pairs, respectively (Table S5). The comparison shows that for both the training and testing data, the EADpred has a higher agreement than the Liao-like method, indicating that our method is better than previous site-based methods in predicting antigenic variants.

**SI References**

1. Archetti I & Horsfall FL, Jr. (1950) Persistent antigenic variation of influenza A viruses after incomplete neutralization in ovo with heterologous immune serum.J Exp Med 92(5):441-462.

2. Ndifon W, Dushoff J, & Levin SA (2009) On the use of hemagglutination-inhibition for influenza surveillance: surveillance data are predictive of influenza vaccine effectiveness.Vaccine 27(18):2447-2452.

3. Bao Y, et al. (2008) The influenza virus resource at the National Center for Biotechnology Information. J Virol 82(2):596-601.

4. Larkin MA, et al. (2007) Clustal W and Clustal X version 2.0. Bioinformatics 23(21):2947-2948.

5. National Center for Health Statistics (2009) Vital Statistics Data Available Online, Mortality Multiple Causes Files, 1968-2006. Available on [http://www.cdc.gov/nchs/data_access/Vitalstatsonline.htm#Mortality_Multiple](http://www.cdc.gov/nchs/data_access/Vitalstatsonline.htm#Mortality_Multiple).

6. CDC. 122 Cities Mortality Reporting System. Available on <http://wonder.cdc.gov/mmwr/mmwrmort.asp>.

7. U.S.Census Bureau, Population estimate. Available on <http://www.census.gov/popest/cities/cities.html>.

8. Couch RB & Kasel JA (1983) Immunity to influenza in man. Annu Rev Microbiol 37:529-549.

9. CDC. 2001-02 influenza season summary. Available on <http://www.cdc.gov/flu/weekly/weeklyarchives2001-2002/01-02summary.htm>.

10. Lee MS & Chen JS (2004) Predicting antigenic variants of influenza A/H3N2 viruses. Emerg Infect Dis 10(8):1385-1390.

11. Liao YC, Lee MS, Ko CY, & Hsiung CA (2008) Bioinformatics models for predicting antigenic variants of influenza A/H3N2 virus. Bioinformatics 24(4):505-512.

12. Gerhard W, Yewdell J, Frankel ME, & Webster R (1981) Antigenic structure of influenza virus haemagglutinin defined by hybridoma antibodies. Nature 290(5808):713-717.

13. Caton AJ, Brownlee GG, Yewdell JW, & Gerhard W (1982) The antigenic structure of the influenza virus A/PR/8/34 hemagglutinin (H1 subtype). Cell 31(2 Pt 1):417-427.

14. Reid AH, Fanning TG, Hultin JV, & Taubenberger JK (1999) Origin and evolution of the 1918 "Spanish" influenza virus hemagglutinin gene. Proc Natl Acad Sci U S A 96(4):1651-1656.

15. Kulkarni-Kale U, Bhosle S, & Kolaskar AS (2005) CEP: a conformational epitope prediction server. Nucleic Acids Res 33(Web Server issue):W168-171.

16. Hubbard SJ & Thornton JM (1993) 'NACCESS', Computer Program, Department of Biochemistry and Molecular Biology, University College London.

17. Ndifon W, Wingreen NS, & Levin SA (2009) Differential neutralization efficiency of hemagglutinin epitopes, antibody interference, and the design of influenza vaccines. Proc Natl Acad Sci U S A 106(21):8701-8706.

18. Ekiert DC, et al. (2009) Antibody recognition of a highly conserved influenza virus epitope. Science 324(5924):246-251.

19. Gupta V, Earl DJ, & Deem MW (2006) Quantifying influenza vaccine efficacy and antigenic distance. Vaccine 24(18):3881-3888.

20. Lee MS, Chen MC, Liao YC, & Hsiung CA (2007) Identifying potential immunodominant positions and predicting antigenic variants of influenza A/H3N2 viruses. Vaccine 25(48):8133-8139.

21. Kawashima S, et al. (2008) AAindex: amino acid index database,

progress report 2008. Nucleic Acids Res 36, (Database issue):D202-205.
